# Uncertainty‐Quantified Primary Particle Size Prediction in Li‐Rich NCM Materials via Machine Learning and Chemistry‐Aware Imputation

**DOI:** 10.1002/advs.202515694

**Published:** 2025-10-08

**Authors:** Benediktus Madika, Chaeyul Kang, JooSung Shim, Taemin Park, Jung Hyeon Moon, EunAe Cho, Seungbum Hong

**Affiliations:** ^1^ Department of Materials Science and Engineering KAIST Daejeon 34141 South Korea; ^2^ KAIST Institute for NanoCentury (KINC) KAIST Daejeon 34141 South Korea

**Keywords:** chemistry‐aware imputation, lithium‐rich, natural gradient boosting, primary particle size, probabilistic machine learning, sintering conditions, uncertainty quantification

## Abstract

Lithium‐rich Nickel–Cobalt–Manganese (Li‐rich NCM) materials are promising cathodes for lithium‐ion batteries, where electrochemical performance is sensitive to primary particle size. Yet, efforts to apply machine learning (ML) for particle size prediction are constrained by incomplete literature data. This study applies imputation methods—MatImpute, K‐nearest neighbors, multivariate imputation by chained equations, and Mean—to complete the datasets, followed by training a Natural Gradient Boosting (NGBoost) model to predict primary particle size with quantified uncertainty. Two training strategies are evaluated: one including entries with imputed target values and another excluding them. Both strategies are tested on a fully observed dataset. The MatImpute‐based NGBoost model achieves the highest accuracy, with a test R^2^ of 0.866 and a calibration error of 0.133. Feature analysis identifies second sintering temperature and first sintering time as dominant factors, with composition showing minimal influence, consistent with prior experimental reports that sintering parameters drive sub‐micron grain growth through atomic mobility and grain coarsening. Experimental validation shows most predictions within 0.13 µm of measurements and normalized uncertainties near 1.5. These findings demonstrate that robust imputation and uncertainty quantification enhance ML‐based particle size prediction and confirm that sintering conditions, rather than stoichiometry, govern microstructural evolution in Li‐rich NCM materials.

## Graphical abstract

This study demonstrates a machine learning framework that predicts the primary particle size of Lithium‐rich Nickel‐Cobalt‐Manganese (Li‐rich NCM) materials from synthesis conditions, even with incomplete literature data. By combining chemistry‐aware imputation with uncertainty‐quantified modeling, it identifies sintering temperature and time as dominant factors, enabling more reliable, data‐driven optimization of microstructure for high‐performance lithium‐ion batteries.

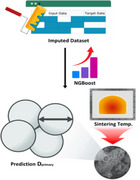

## 1 Introduction

Lithium‐rich Nickel–Cobalt–Manganese (Li‐rich NCM) materials are among the most promising classes of cathodes for achieving high energy density in next‐generation lithium‐ion batteries (LIBs).^[^ ^1^, ^2^, ^3^, ^4^ ^]^ The term “Li‐rich” refers to compositions with excess Li in the transition metal (TM) layer, typically expressed as Li_1+δ_​(Ni,Co,Mn)_1−δ_​O_2_, where δ denotes the fraction of Li occupying the TM layer. At the microstructural level, Li‐rich NCM structures consist of alternating TM and lithium layers arranged in a hexagonal framework and enclosed by face‐sharing oxygen octahedra, with primary particles often forming secondary particles through agglomeration into randomly oriented, densely packed polycrystalline aggregates.^[^ ^5^ ^]^

Since primary particle size strongly influences electrochemical properties, surface stability, and mechanical integrity, it is widely regarded as one of the key design parameters for all NCM materials‐based cathodes of LIBs.^[^ ^6^, ^7^ ^]^ Mainly controlled by sintering parameters (e.g., temperature, time) and chemical composition, primary particle size strongly dictates electrochemical behavior: smaller grains improve Li‐ion transport and kinetics but increase surface reactivity, whereas larger grains mitigate side reactions but impede transport and accelerate voltage fade.^[^ ^8^, ^9^, ^10^, ^11^, ^12^ ^]^ Therefore, developing a quantitative approach that links synthesis parameters and composition to primary particle size is essential for optimizing Li‐rich NCM cathode materials in LIBs. Machine learning (ML) enables quantitative modeling by capturing the complex, nonlinear relationships between synthesis parameters and composition, and the resulting primary particle size, thereby accelerating the design and optimization of Li‐rich NCM materials.^[^ ^13^ ^]^

However, applying ML to this problem remains challenging because experimental reports on Li‐rich NCM materials often contain incomplete or inconsistent data. While sintering parameters and chemical composition (the input variables for the ML model) are typically well documented, primary particle size (the target variable of the ML model) is frequently missing or only described qualitatively (e.g., “sub‐µm”), limiting the construction of reliable datasets for ML predictive modeling. Conventional materials informatics workflows typically discard data entries with missing data in the target variable, which can significantly reduce dataset size. For example, previous research reported that ≈ 60% of property fields in organic‐materials databases were missing, and this lack of completeness demonstrated that list‐wise deletion could severely reduce ML predictive performance.^[^ ^14^ ^]^ Similarly, in an LIB cathode synthesis study, removing incomplete entries reduced the dataset size, which can drastically limit the amount of training data available and impair model accuracy.^[^ ^15^ ^]^ A potential alternative is to retain such entries by substituting the missing target values with estimated ones for training purposes only, while restricting evaluation to complete entries, thereby preserving dataset size without compromising test reliability.

To address missing data in the materials science domain, a recent study has demonstrated the effectiveness of a chemistry‐aware imputation method called MatImpute, which outperformed other imputation methods when benchmarked on imputing various chemistries of materials for ML predictions.^[^ ^16^ ^]^ MatImpute initiates tentative values using K‐nearest neighbors (KNN)‐based row‐wise estimation and then employs Extra Trees (ET)‐based column‐wise imputation, iteratively updating the dataset until all missing entries are filled. This approach better preserves value distributions and feature correlations than generic imputations, such as Mean, KNN, and multiple imputations by chained equations (MICE).

Nevertheless, imputing missing data alone represents only a partial solution, as it introduces additional uncertainty that must be accounted for during model training and evaluation, while intrinsic variability in experimental measurements further compounds the overall uncertainty. Most existing studies in materials science, however, employ deterministic ML models, such as Random Forests for corrosion rate prediction,^[^ ^17^ ^]^ Gradient Boosting for molecular property prediction,^[^ ^18^ ^]^ or Multilayer Perceptrons for predicting the mechanical property of carbon fiber,^[^ ^19^ ^]^ which provide only point estimates without quantifying prediction reliability. Probabilistic ML provides a more suitable framework by delivering both point predictions and calibrated uncertainty estimates, and it also captures both aleatoric (data‐related) and epistemic (model‐related) uncertainties.^[^ ^20^ ^]^ Therefore, it can propagate imputation‐related uncertainty, capture intrinsic experiment and measurement variability, and support downstream applications such as synthesis planning or inverse design, where confidence estimates are essential.^[^ ^21^, ^22^ ^]^ The quality of these uncertainty estimates can be assessed using metrics such as the area under calibration error (AUCE) and negative log‐likelihood (NLL), and coefficient of determination (R^2^).^[^ ^20^ ^]^ Among available probabilistic ML models, Natural Gradient Boosting (NGBoost), introduced by the Stanford ML Group reported a promising approach for structured tabular data, offering flexibly calibrated distributional outputs.^[^ ^23^, ^24^ ^]^ However, its potential remains underexplored in the context of Li‐rich NCM materials systems, particularly for modeling the relationship between sintering parameters and primary particle size in the presence of imputed and noisy experimental data.

Building on this motivation, we developed the NGBoost‐based probabilistic ML model to predict the mean primary particle size of Li‐rich NCM materials from composition and the four sintering‐related process variables—first sintering temperature, first sintering time, second sintering temperature, and second sintering time (hereafter referred to collectively as sintering parameters). To enable data‐driven prediction under real‐world experimental sparsity, we imputed the Li‐rich NCM materials dataset using four imputation strategies, namely MatImpute, KNN, MICE, and Mean, before using it to train the NGBoost model. Unlike Xie et al., who benchmarked MatImpute primarily on generic datasets using statistical fidelity metrics,^[^ ^16^ ^]^ our study applies MatImpute as a chemistry‐aware imputation method to impute missing input values (*X*) and missing target values (*y*) in the context of Li‐rich NCM materials, thereby enabling the construction of a more complete dataset for uncertainty‐quantified probabilistic prediction of primary particle size.

To assess the impact of the dataset completeness on the ML model, we compared two training strategies: i) training NGBoost on datasets that included data entries with imputed missing target values (here, the dataset is referred as imputed *X*−*y* dataset), thereby utilizing both complete and incomplete data entries, and ii) training NGBoost exclusively on datasets that included only data entries with fully observed target values (here, the dataset is referred as imputed *X* dataset), discarding any data entries with missing target values. In both strategies, model performance was evaluated on a complete held‐out test set (with no missing input (*X*) or target values (*y*) to ensure a fair comparison.

Since experimental data on Li‐rich NCM materials are inherently noisy due to intrinsic experimental variability and missing data, and are further complicated by imputation‐induced uncertainty, these factors collectively underscore the need for probabilistic predictions and explicit quantification of uncertainty during model training and evaluation. Therefore, we trained the NGBoost model on each imputed dataset and evaluated its overall probabilistic performance (NLL), accuracy (R^2^), and calibration (AUCE) on both the training and complete held‐out test sets. To examine robustness under realistic deployment conditions, we conducted out‐of‐distribution (OOD) evaluation using LiNi_0.8_Co_0.1_Mn_0.1_O_2_ (NCM811) samples, which, unlike Li‐rich NCM that contains excess Li in the TM layer (δ  >  0), are Ni‐rich layered oxides with δ  =  0. This compositional and structural distinction makes NCM811 a suitable OOD test case, as it belongs to the same NCM family but lies outside the Li‐rich compositional space represented in the training data.

This study demonstrates that the NGBoost model, trained on the MatImpute‐imputed *X* − *y* dataset, achieves the highest accuracy and strongest overall performance. SHapley Additive exPlanations (SHAP) reveal that the second sintering temperature and the first sintering time dominate primary particle size prediction, consistent with established synthesis–structure–property relationships. OOD testing confirms that the NGBoost model not only maintains accuracy but also preserves uncertainty estimates, demonstrating the robustness of our framework. By integrating chemistry‐aware imputation, probabilistic ML, and uncertainty quantification, this work enables reliable primary particle size prediction in Li‐rich NCM materials, even with incomplete data, while ensuring calibrated predictions.

## 2 Results

**Figure 1:**
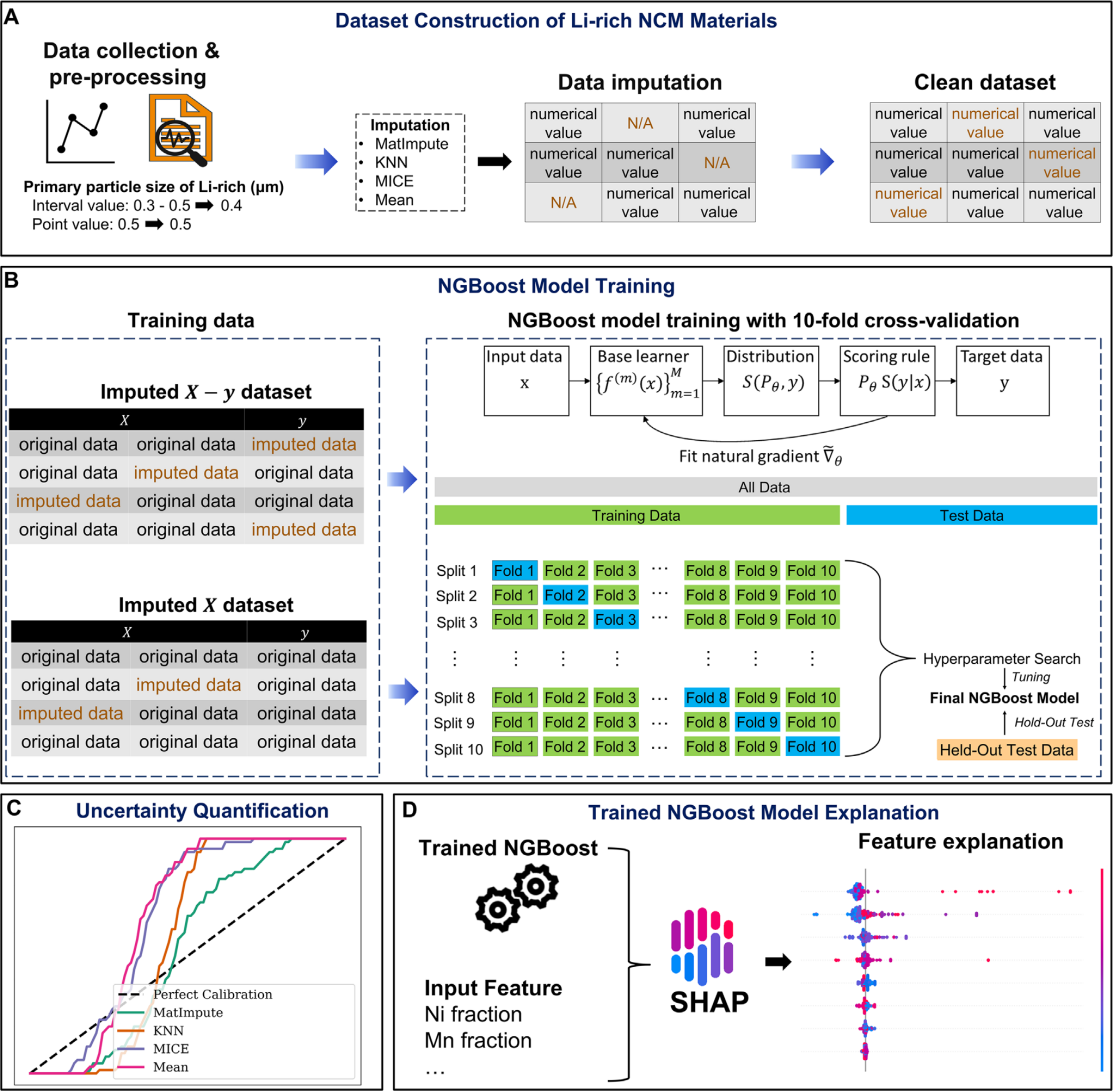
General workflow. A) Data pre‐processing schematic including standardization and imputation methods. B) The NGBoost training pipeline employing 10‐fold cross‐validation, comparing two approaches: i) imputing data entries, including those with missing target values (imputed *X*−*y* dataset), and ii) discarding data entries with missing target values before imputation (imputed *X* dataset). C) UQ via AUCE for different imputation methods. D) SHAP analysis.

As outlined in the Experimental Section, primary particle sizes reported as intervals in the literature were converted to their mean value to enable their use in the NGBoost prediction. All missing data entries were then addressed using four imputation strategies, creating complete datasets for subsequent model training (**Figure** **1**A). As shown in Figure 1B, the NGBoost model was trained using imputed datasets handled in two strategies: i) imputing data entries including those with missing target values (the resulting data is denoted as imputed X−y dataset with 293 data points), and ii) discarding data entries with missing target values before imputation (the resulting data is denoted as imputed X dataset with 143 data points).

Both models in the two data imputation strategies were trained using 10‐fold cross‐validation, with input variables including the elemental fractions of Li in the TM layer, Ni, Co, and Mn, as well as first and second sintering temperatures and times. The NGBoost model's performance was evaluated on a complete held‐out test (with no missing data in either the input (*X*) or target (*y*) variables), ensuring a fair assessment of generalization. The model's predictive performance was evaluated using three metrics: NLL, accuracy (R^2^), and AUCE for calibration. AUCE captures the alignment between predicted confidence intervals and actual values (Figure 1C). Finally, SHAP analysis was used to interpret the influence of individual input features on the NGBoost model's prediction of primary particle size (Figure 1D).

### 2.1 Analysis of the NGBoost Model Trained on the Imputed Dataset

Four imputation methods—MatImpute, KNN, MICE, and Mean—were applied to impute missing data in both data‐handling strategies mentioned above. Model performance was assessed using 10‐fold cross‐validation, with evaluation based on R^2^ and NLL metrics.

As shown in **Tables** **1** and **2**, the NGBoost model trained on the MatImpute‐imputed *X*−*y* dataset, yielded the highest performance (R^2^  =  0.88, NLL  =  −0.79), indicating the strongest accuracy and well‐calibrated uncertainty among others. In contrast, the NGBoost models trained on the KNN‐ and MICE‐imputed *X*−*y* datasets showed moderate predictive accuracy values (R^2^ values were 0.49 and 0.58, respectively), but poor calibration (NLL values were −0.21 and 0.14, respectively), while the one trained on the Mean‐imputed *X*−*y* dataset consistently underperformed (R^2^  =  −1.31, NLL  =  −0.08).

**Table 1:** NLL values for each fold of the NGBoost model trained on the imputed *X*−*y* datasets.

Imputed *X*−*y* Datasets	Fold										
	1	2	3	4	5	6	7	8	9	10	Average NLL
MatImpute‐imputed dataset	−0.87	−0.82	−1.29	−0.64	−1.10	−0.74	−0.13	−0.68	−1.09	−0.49	−0.79
KNN‐imputed dataset	2.95	−0.57	−1.09	−0.48	−0.81	−0.41	−0.12	−0.66	−0.65	−0.23	−0.21
MICE‐imputed dataset	0.88	−0.67	−0.69	0.56	−0.10	−0.18	−0.27	0.30	−0.71	2.27	0.14
Mean‐imputed dataset	1.67	−0.61	−0.63	−0.30	0.23	−0.14	−0.19	−0.36	−0.45	−0.03	−0.08

**Table 2:** The R^2^ values for each fold of the NGBoost model trained on the imputed *X*−*y* datasets.

Imputed *X*−*y* datasets	Fold										
	1	2	3	4	5	6	7	8	9	10	Average R^2^
MatImpute‐imputed dataset	0.91	0.98	0.33	0.88	0.98	0.92	0.89	0.95	0.97	0.96	0.88
KNN‐imputed dataset	0.69	0.96	−2.83	0.85	0.94	0.87	0.89	0.68	0.92	0.97	0.49
MICE‐imputed dataset	0.73	0.96	0.10	0.27	0.60	0.79	0.92	−0.44	0.95	0.87	0.58
Mean‐imputed dataset	0.73	0.96	−0.40	0.18	−7.87	0.81	0.91	−10.2	0.92	0.90	−1.31

The NGBoost models performed significantly worse when trained on imputed *X* datasets (Tables S1 and S2, Supporting Information). Averaged NLL values increased to 0.89–1.00, indicating poor uncertainty calibration, while averaged R^2^ values dropped as low as − 1.27, reflecting poor predictive performance. These findings highlight the importance of retaining as much data as possible and leveraging chemistry‐aware imputation to ensure robust, uncertainty‐quantified predictions.

Table S3 (Supporting Information) reports averaged cross‐validation scores for a random 143‐row subsample drawn from the 293‐data entry imputed *X*−*y* datasets. This subsample was used to ensure the same data size for a fair comparison with the 143‐data entry imputed *X* datasets in Tables S1 and S2 (Supporting Information), which contain no missing target values. Although the number of data points is the same, differences in data composition influence the performance patterns. Shifting from datasets without missing data in the target variable to datasets with and without missing target values results in a decrease in the NGBoost model accuracy (R^2^). This can be seen when the NGBoost was trained on MatImpute‐, KNN‐, or Mean‐imputed datasets, but an improvement was seen when trained on the MICE‐imputed dataset. In contrast, NLL improves for models trained on MatImpute‐, MICE‐, and Mean‐imputed datasets, but worsens with the KNN‐imputed dataset. These results highlight that, beyond sample size, the composition of the training data significantly affects both prediction accuracy and uncertainty behavior.

As illustrated in the violin plots in Figure S1 (Supporting Information), the distributions of input variables reveal significant performance differences among imputation methods. Stoichiometric features, such as elemental fractions, had no missing data and exhibited consistent distributions across all imputation strategies. However, features with missing data—particularly sintering conditions and mean primary particle size—were notably affected by imputation, leading to substantial distortions. The exclusion of missing target values in the original dataset introduced bias, skewing the distribution of stoichiometric variables. The Mean imputation reduced data variability by compressing feature spread, while KNN and MICE imputations distorted the plausibility of process parameters. In contrast, MatImpute best preserved the original variability. Features such as second sintering time and primary particle size retained their distributions, with only minor shifts in tail behavior. The close alignment of the median and interquartile range in the MatImpute‐imputed *X*−*y* dataset with the original data confirms its minimal impact on data integrity. The original target data were strongly right‐skewed, with most values in the 0–1 µm range and a sparse tail up to ≈ 5 µm (Figure S2A, Supporting Information). After applying MatImpute, the distribution shape was maintained, with imputed missing values concentrated in the sub‐micron range, without introducing artificial extremes (Figure S2B, Supporting Information). In contrast, the dataset without missing target values showed reduced coverage due to the removal of rows with missing data (Figure S2C, Supporting Information). This faithful preservation of variability likely contributed to the enhanced predictive performance observed in models trained on the MatImpute‐imputed *X*−*y* dataset, reinforcing its effectiveness in maintaining both physical realism and statistical robustness during imputation.

Correlation analyses (Figures S3 and S4, Supporting Information) demonstrated that all imputation methods consistently preserved underlying correlations among explanatory variables. The robustness of these intrinsic feature relationships indicated that observed predictive performance variations primarily resulted from individual feature distribution rather than the fundamental inter‐feature relationships.

**Figure 2:**
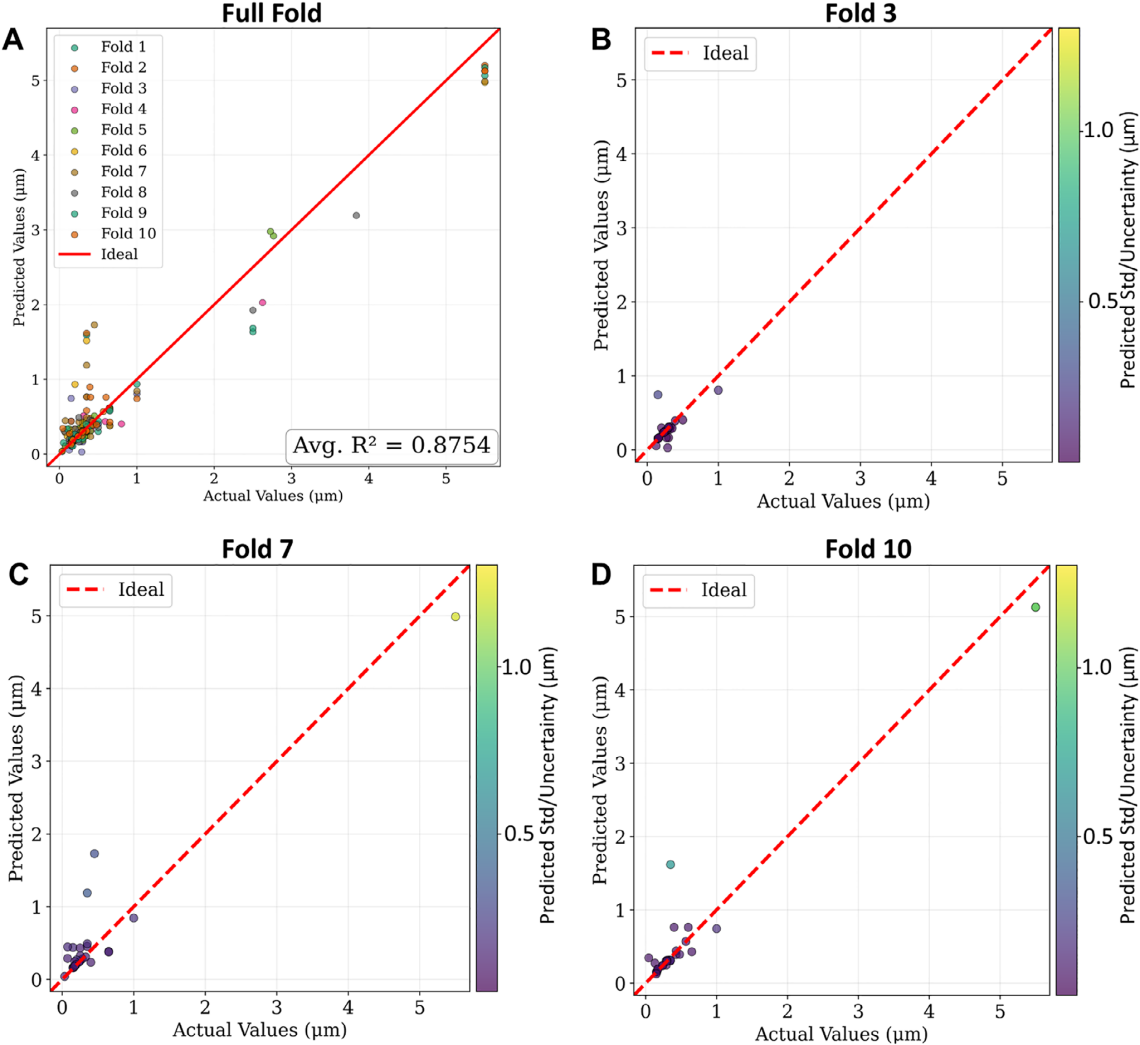
Performance of the NGBoost model trained on the MatImpute‐imputed *X*−*y* dataset. A) Aggregate parity plot across all ten folds showing strong overall agreement (average R^2^ = 0.8754). B–D) Representative folds chosen for their contrasting R^2^ and NLL values in Tables 1 and 2: Fold 3 shows the lowest deviations and best calibration despite modest R^2^, Fold 10 demonstrates intermediate deviations with occasional overconfidence, and Fold 7 exhibits the largest deviations at large particle sizes but appropriately high uncertainties (std is the standard deviation).

**Figure** **2**A shows the aggregate parity plot across all ten folds, with predictions clustered near the diagonal generally associated with lower predicted standard deviations. Figure 2B–D highlight folds 3, 7, and 10, selected from the 10‐fold cross‐validation runs based on their contrasting R^2^ and NLL values in Tables 1 and 2. The three folds highlighted (3, 7, and 10) were deliberately chosen because they represent combinations of R^2^ and NLL, which deviated from the average behavior. Fold 3 showed the lowest deviations and best calibration (above‐average NLL (− 1.29)) despite modest R^2^ (below‐average R^2^ (0.33))—an example where probabilistic performance remains strong even with modest variance explained. Fold 10 demonstrated intermediate deviations with occasional miscalibration (below‐average NLL (− 0.49)) but with above‐average R^2^ (0.96). Fold 7 displayed the largest deviations and the highest predicted uncertainty (worst NLL (− 0.13)), reflecting data sparsity with an average R^2^ (0.89). Figure S5 (Supporting Information) illustrates representative folds selected from the remaining cross‐validation runs. Fold 2 (R^2^ =  0.98, NLL =   − 0.82) shows behavior closely aligned with that of Fold 10, achieving near‐ideal parity in the sub‐micron regime but incurring a calibration penalty driven by a single large‐size data point. By contrast, Fold 4 (R^2^ = 0.88, NLL =   − 0.64) mirrors the trend observed in Fold 3, where a slight reduction in accuracy is offset by improved probabilistic calibration across the particle‐size range.

The deviation ranking, Fold 3 <  Fold 10 <  Fold 7, illustrates a progression from well‐calibrated low‐error predictions to high‐error but uncertainty‐quantified predictions. Examining these folds is crucial because it shows that no single performance metric captures full model behavior; calibration and accuracy must be considered together. This interpretation is further supported by the training‐loss trajectories in Figure S6 (Supporting Information), which display both the raw logged losses and the exponentially weighted moving average (EWMA)–smoothed trends. Fold 3 achieved the most stable convergence, with consistently decreasing raw curves and the lowest final validation loss, accompanied by only a minimal train–validation gap. Fold 10 showed intermediate stability, where the raw curves fluctuated moderately but the smoothed trend confirmed steady convergence. By contrast, Fold 7 converged more weakly, with noisier raw losses and a larger train–validation gap, even after smoothing. Together, these results demonstrate that folds 3, 7, and 10 provide a representative deviating insight into how the model balances accuracy and calibration under different data distributions and training dynamics.

Training stability and convergence were examined through the loss trajectories presented in Figure S7 (Supporting Information). For all 10‐fold‐cross‐validation folds, both training and validation NLL values exhibited consistent decreases, with the most pronounced improvements occurring in the early stages of training. Early stopping, governed by validation NLL, resulted in several folds terminating before the 300‐iteration limit once further improvement was no longer observed. Across folds, validation losses stabilized without subsequent rebound, and the gaps between training and validation curves remained small, satisfying the predefined criteria for a good fit and indicating minimal overfitting. The final NGBoost model trained on the MatImpute‐imputed *X*−*y* dataset displayed convergence dynamics that closely paralleled those of the cross‐validation folds.

### 2.2 Test Performance of the NGBoost Model Trained on the MatImpute‐Imputed *X*−*y* Dataset

**Figure 3:**
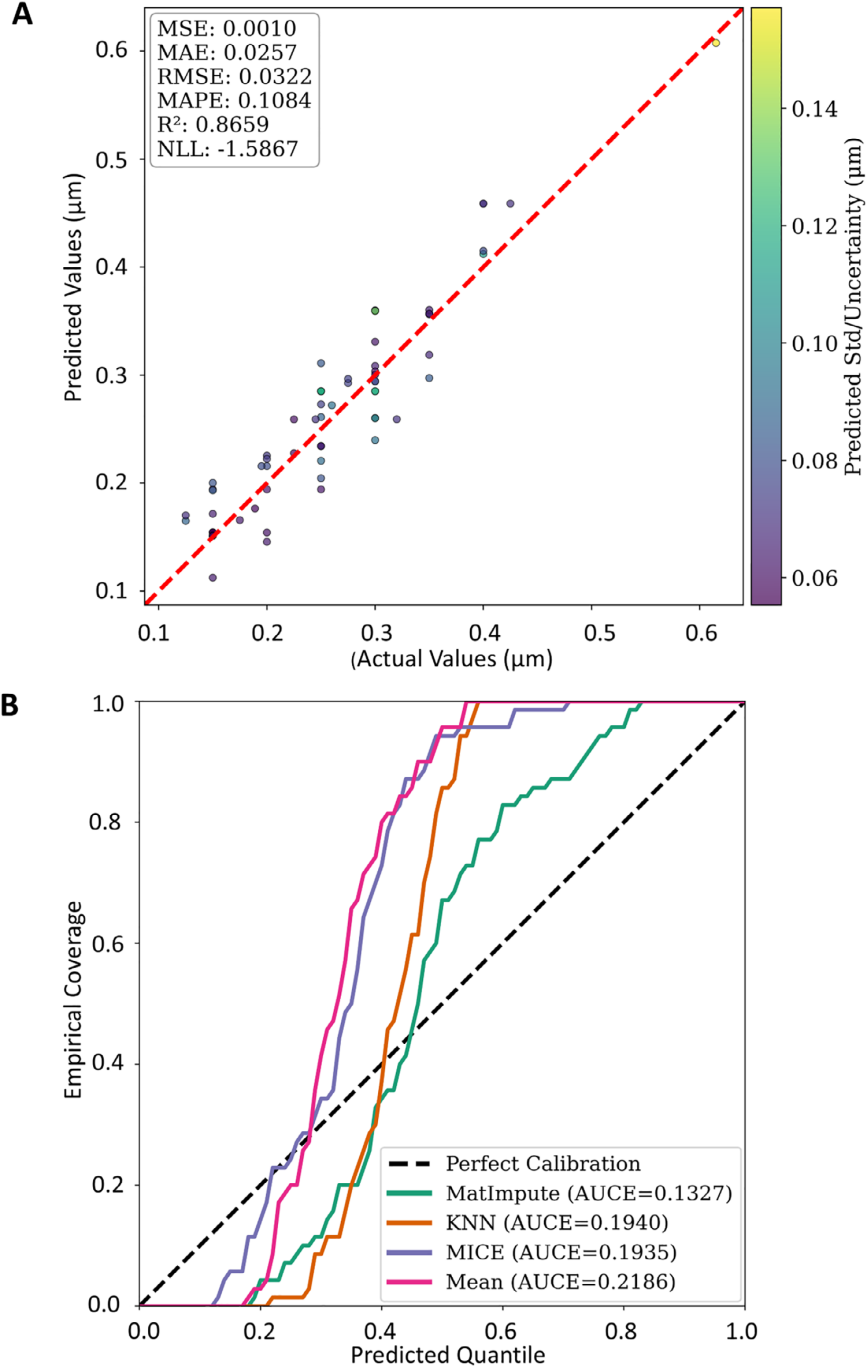
NGBoost performance on a complete held‐out test set. A) Parity plot comparing predicted and actual primary particle sizes, with prediction uncertainty/std represented by color intensity. B) Calibration accuracy comparison using the AUCE metric of the NGBoost models trained on the datasets, including data entries with missing target values, imputed using various imputation methods: MatImpute, KNN, MICE, and Mean. MatImpute provided the best calibration performance, with the lowest AUCE (0.1327).

**Figure** **3** provides an evaluation of NGBoost performance when trained on the MatImpute‐imputed *X*−*y* dataset, showcasing both its predictive accuracy and uncertainty calibration. In Figure 3A, the parity plot demonstrates that predicted means closely align with actual values across the entire range of mean primary particle sizes, clustering around the diagonal line with no evident skew or systematic bias. The minimal scatter indicates that the model generalizes well, even for mid‐ to high‐size ranges. The color bar, representing model‐predicted uncertainty (standard deviation, std), shows that prediction confidence varies with sample difficulty—darker purple points (lower uncertainty) are concentrated along the diagonal, while lighter green‐yellow points (higher uncertainty) appear farther from the diagonal, indicating the model's awareness of outlier‐like predictions. The statistical inset (MSE = 0.0010, MAE =  0.0257 µm, RMSE =  0.0322 µm, R^2^  = 0.8659, NLL =   − 1.5867) confirms that the model exhibits low error and sharp, well‐calibrated predictive distributions, further emphasizing its robust performance.

Figure 3B further validates the results by comparing empirical coverage against predicted quantiles across four imputation strategies. The MatImpute curve is closest to the perfect calibration line (45° dashed), particularly in the critical quantile range between 0.3 and 0.8, where high data density typically resides. While a slight dip below the diagonal is observed in the 0.2–0.4 quantile range, suggesting minor overconfidence, this deviation is much smaller when compared to the KNN, MICE, or Mean imputation strategies. The AUCE metric quantifies this calibration performance: MatImpute (0.1327) outperforms KNN (0.1940), MICE (0.1935), and Mean (0.2186), with a clear gap in performance. Notably, the Mean curve plateaus early and consistently underperforms across all quantiles, indicating poor uncertainty estimation regardless of the predicted confidence levels.

These findings confirm that MatImpute is not only the most effective imputation method for minimizing prediction error but also the most reliable for generating trustworthy predictive intervals. By combining close parity, adaptive uncertainty estimation, and a near‐ideal calibration curve, MatImpute emerges as the most robust pre‐processing strategy for primary particle size prediction in datasets with missing values in this study.

### 2.3 The NGBoost Model Interpretation, Trained on the MatImpute‐Imputed *X*−*y* Dataset, Using SHAP

**Figure 4:**
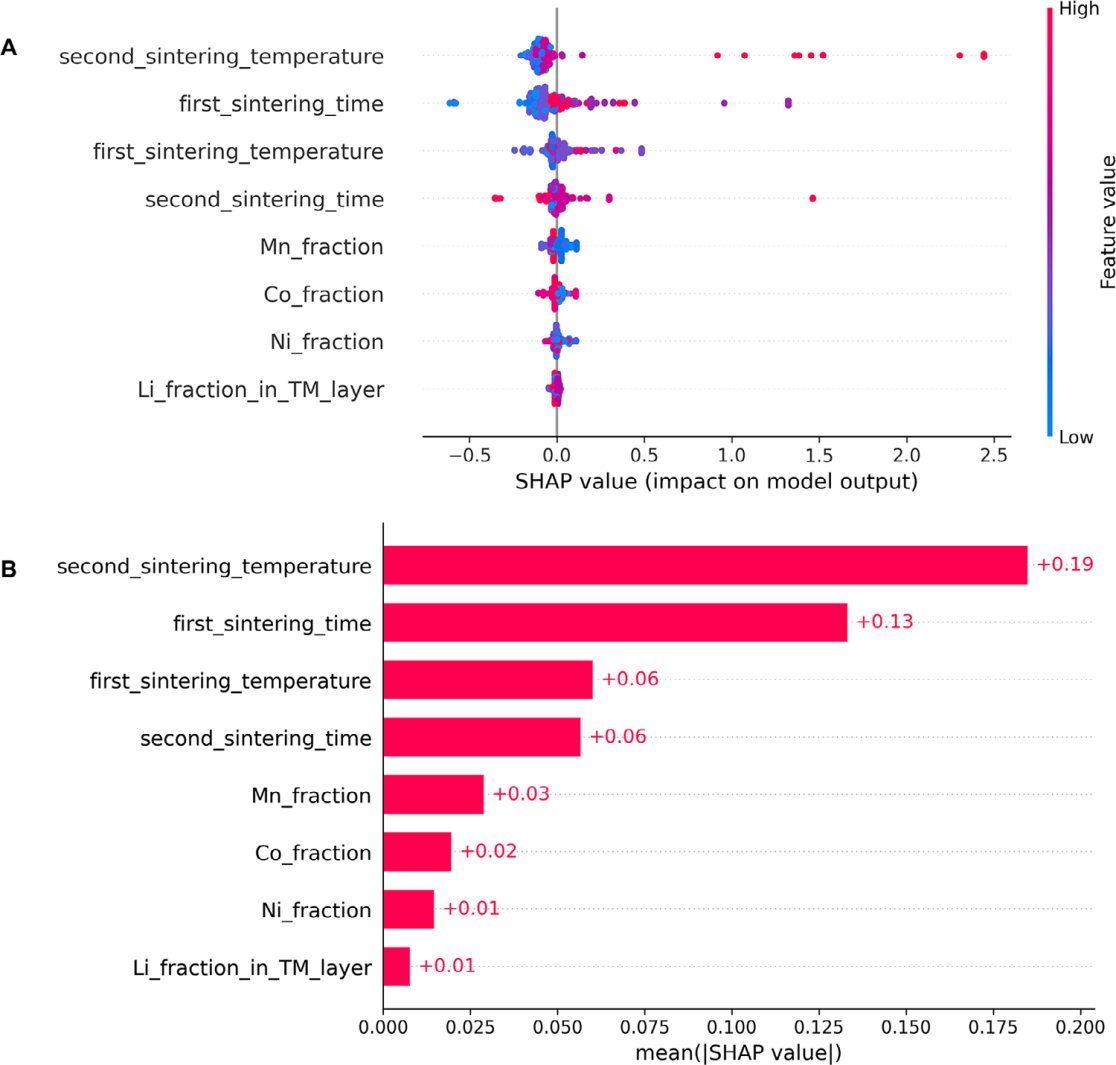
SHAP analysis of NGBoost predictions trained on the MatImpute‐imputed *X*−*y* dataset. A) SHAP summary plot showing the effect of individual features on predicted particle size. The second sintering temperature has the strongest influence, followed by the first sintering time, while compositional fractions (Mn, Co, Ni, Li in TM layer) have minimal impact. B) Mean absolute SHAP values quantify global feature importance, confirming the second sintering temperature as the most dominant feature, followed by the first sintering time.

**Figure** **4**A presents a SHAP summary plot illustrating the contribution of each feature to mean primary particle size predictions, where the color indicates the feature value and the horizontal position reflects the impact relative to the global mean. The second sintering temperature stands out as the most influential parameter, where higher values consistently lead to larger predicted particle sizes. This trend is further supported by the global SHAP importance rankings in Figure 4B, which identify second sintering temperature and first sintering time as the top predictors based on mean absolute SHAP values. In contrast, the first sintering temperature and second sintering time exhibit more moderate and less consistent contributions. Compositional variables, including Mn, Co, Ni, and Li in TM layer fractions, show minimal influence, with SHAP values concentrated near zero across all samples, confirming that stoichiometry plays a limited role in determining primary particle size within this dataset.

**Figure 5:**
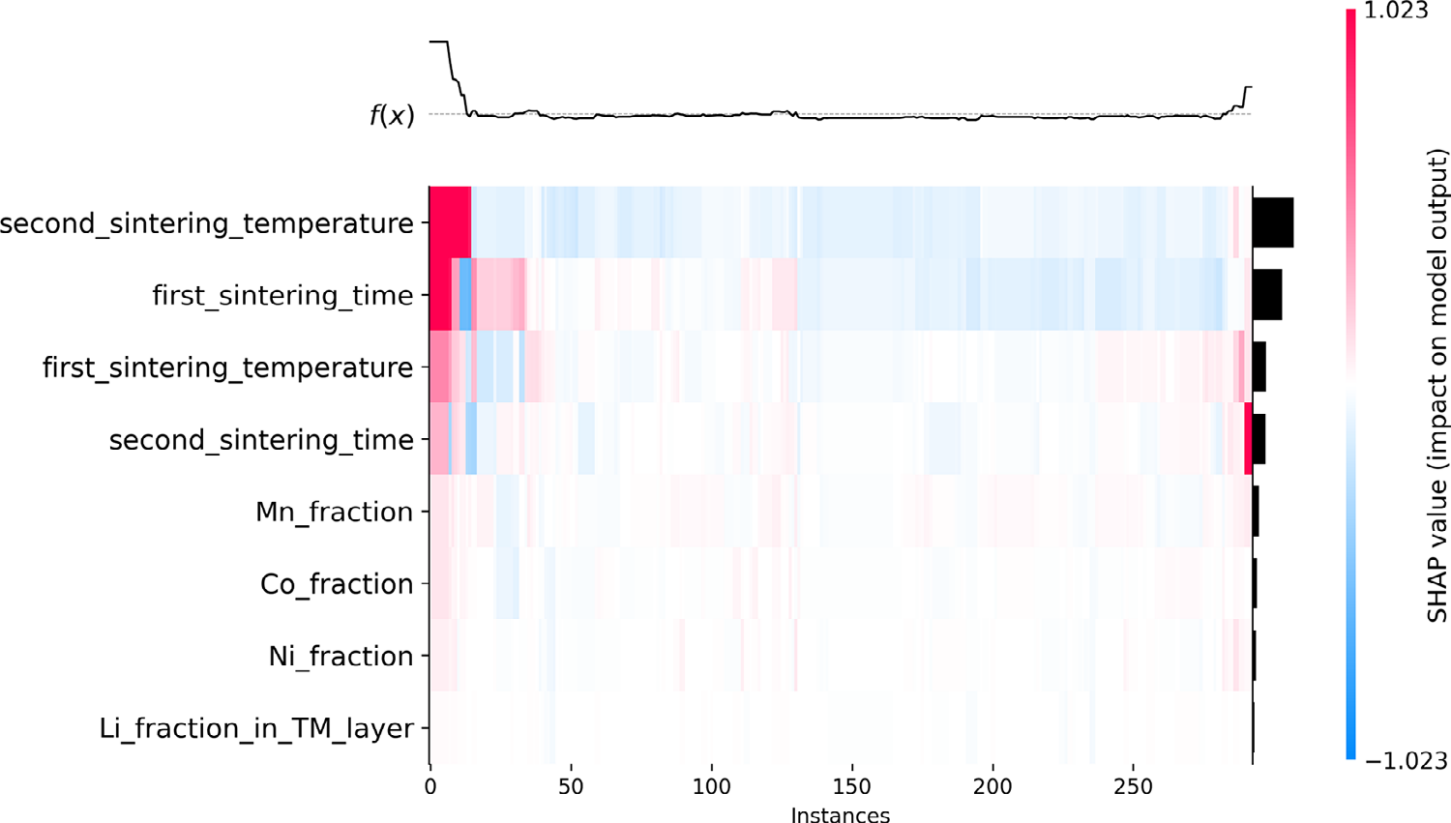
SHAP per‐instance decision analysis of NGBoost trained on the MatImpute‐imputed *X*−*y* dataset. The dashed line marks the mean model output (baseline), with curves showing deviations explained by SHAP feature contributions. The second sintering temperature is the strongest driver of larger particle size predictions, followed by the first sintering time. Other parameters have moderate effects, while compositional features show minimal influence. The bar plot on the right confirms the dominance of the second sintering temperature and the secondary role of the first sintering time.

To examine coupled effects, SHAP interaction values were analyzed (Figure S8, Supporting Information). Each column represents a source feature and each row a target feature, with the horizontal spread showing the strength of pairwise effects and color indicating whether high (red) or low (blue) values dominate. The widest spreads occur when the source is the second sintering temperature and the targets are the sintering times (first and second), indicating that higher temperatures combined with longer sintering times increase predicted particle size, while lower values decrease it. In contrast, Ni/Co/Mn/Li in TM layer fractions show interactions centered near zero, reflecting a weak influence relative to the sintering parameters. **Figure** **5** further illustrates these effects at the individual sample level using a heatmap of feature contributions. In this plot, the top black curve displays the predicted primary particle sizes *f*(*x*) for each instance, ordered along the x‐axis, with the dashed horizontal line indicating the mean model output (baseline) relative to which SHAP values are computed. The heatmap below shows per‐instance feature contributions, where red indicates a positive impact on particle size predictions and blue indicates a negative impact. It can be seen from the heatmap that the second sintering temperature consistently exerts a strong, positive influence across most instances, while the first sintering time contributes positively in general, albeit with greater variability. Other sintering parameters exhibit mixed and sample‐specific effects, while compositional features remain largely inactive. Overall, both global and local SHAP analyses emphasize that particle size is primarily governed by sintering conditions, underscoring the importance of sintering process optimization over compositional tuning in the synthesis of Li‐rich NCM

### 2.4 Experimental Validation of the NGBoost Model Trained on the MatImpute‐Imputed *X*−*y* Dataset

**Figure 6:**
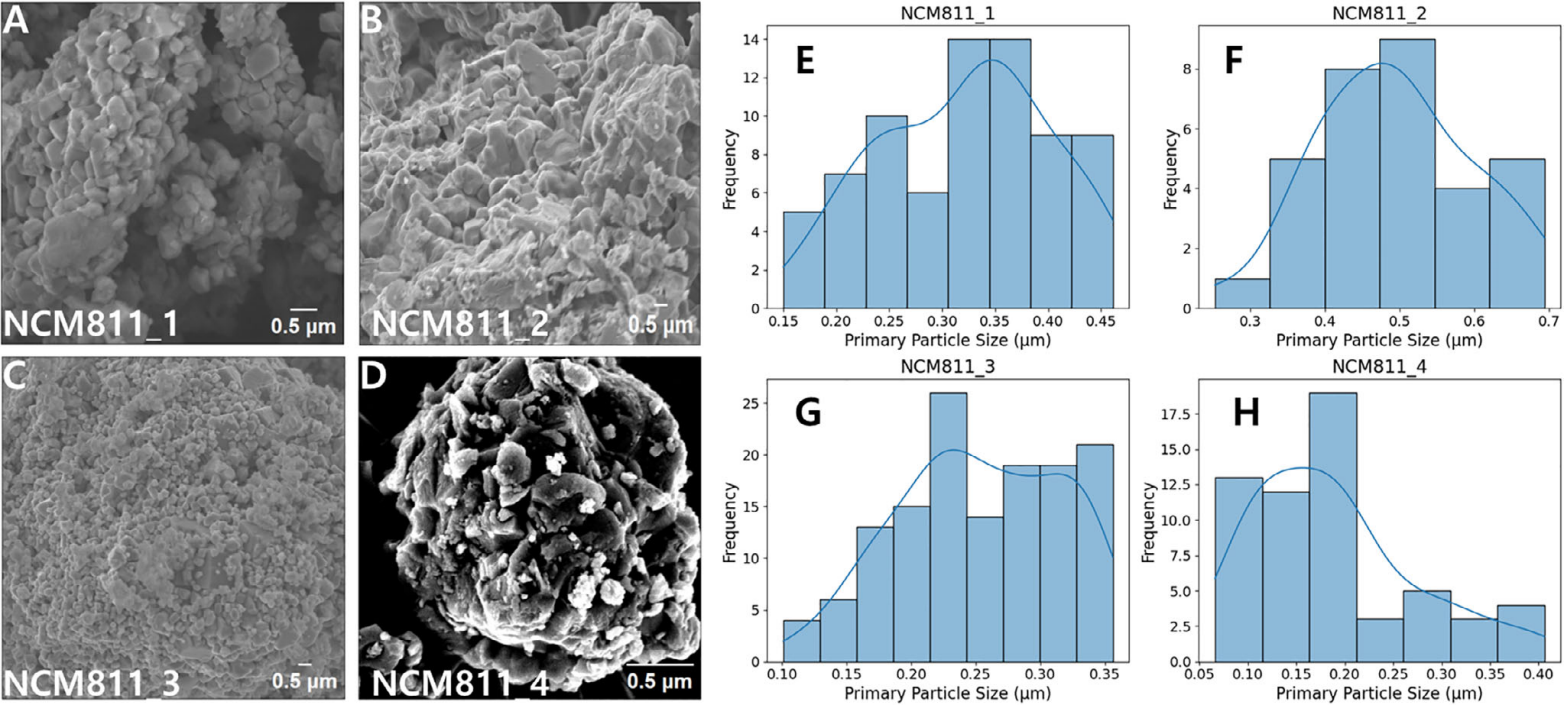
Experimental validation of the NGBoost model, trained on the MatImpute‐imputed *X*−*y* dataset, under distributional shift conditions using four synthesized NCM811 samples. A–D) SEM images showing primary particle morphology for NCM811_1 to NCM811_4, respectively. E–H) Histogram plots of experimentally measured primary particle size distributions for the corresponding samples.

The predictive performance of the NGBoost model, trained on the MatImpute‐imputed *X*−*y* dataset, was evaluated using four experimentally synthesized NCM811 samples, providing an external benchmark to assess its generalizability under distributional shift or OOD. SEM micrographs (**Figure** **6**A–D) highlight morphological differences in the primary particle structures of the samples, while the corresponding histogram plots (Figure 6E–H) depict the particle size distributions extracted from each image. Primary particle sizes were quantified in ImageJ by measuring two orthogonal diameters per particle and averaging the values, with the resulting histograms summarizing these per‐particle measurements. The number of measurements varied across samples: 74 for NCM811_1, 32 for NCM811_2, 136 for NCM811_3, and 58 for NCM811_4. This variation reflects differences in how clearly individual particles could be distinguished and fully recognized in the SEM images.

To evaluate prediction accuracy, the absolute error (∈) was defined as the absolute deviation between the predicted and the measured mean primary particle size. Based on this metric, ∈ values range from 0.024 to 0.131 µm (**Table** **3**), reflecting varying degrees of discrepancy between the predicted and the measured mean particle size. NCM811_4 shows the lowest deviation (∈ ∼0.024 µm), NCM811_1 falls into an intermediate regime (∈ ∼0.115 µm), and NCM811_2 exhibits the largest deviation (∈ ∼0.131 µm). Meanwhile, NCM811_3 has a slightly smaller ∈ (0.130 µm) than that of NCM811_2. However, absolute error alone does not indicate whether the observed differences are statistically meaningful relative to the combined experimental and model uncertainty. For this reason, the normalized error (∈ _Z_), defined in Equation S1 (Supporting Information), was also evaluated. Using this metric, three of the four samples—NCM811_1, NCM811_2, and NCM811_4— exhibit ∈ _Z_ values within the range of |1.10| (− 1.10, − 1.04, and − 0.24, respectively), indicating strong statistical consistency between the predictions and measurements. By contrast, NCM811_3, despite having a slightly smaller ∈ than NCM811_2, exhibits the highest ∈ _*z*_ value (− 1.51), indicating the greatest statistical inconsistency. This outcome reflects residuals that deviate most strongly from the expected distribution and suggests mild overconfidence in the intermediate primary particle size regime (≈ 0.253 µm). Across all samples, the predicted standard deviations (0.0593–0.0740 µm) remain reasonably scaled and correspond to ≈1.5 times the measured standard deviation when normalized by the combined uncertainty, consistent with the overall calibration goal.

**Table 3:** Measured and predicted mean primary particle size for each NCM811 sample, along with its standard deviation and error.

Sample	Measured Mean P_Primary_ [µm]	Measured Std [µm]	Predicted Mean P_Primary_ [µm]	Predicted std [µm]	∈ [µm]	$\in_{Z}$
NCM811_1	0.321	0.0798	0.206	0.0681	0.115	−1.10
NCM811_2	0.495	0.1022	0.364	0.0740	0.131	−1.04
NCM811_3	0.253	0.0625	0.123	0.0593	0.130	−1.51
NCM811_4	0.194	0.0832	0.170	0.0587	0.024	−0.24

P = particle size, std = standard deviation.

## 3 Discussion

This work demonstrates that NGBoost, combined with chemistry‐aware imputation, can accurately and reliably predict primary particle size in Li‐rich NCM materials, even when training data are incomplete. Among the tested imputation strategies, MatImpute preserved realistic variability in synthesis parameters while avoiding implausible values, leading to markedly better accuracy and uncertainty calibration in the prediction model compared with KNN, MICE, and Mean imputation. Retaining incomplete data entries and imputing their missing target values also proved more effective than discarding them, as exclusion reduced sample size and severely degraded both accuracy and calibration.

The close agreement between the mean cross‐validation R^2^ in the training (0.8754) and the test R^2^ (0.8659) on the 293‐sample imputed *X*−*y* dataset indicates that the NGBoost model achieved strong generalization despite the small‐data regime. External validation on synthesized NCM811 samples—produced under conditions outside the training distribution—further confirms that this generalization extends to OOD cases, with uncertainty estimates remaining well‐calibrated. Notably, our OOD validation primarily targeted sub‐micron particles because the synthesis conditions were generated via our in‐house Bayesian optimization workflow. While this limited direct sampling above 1 µm does not represent a weakness, both the training and test distributions are intrinsically concentrated in the sub‐micron domain, with only a small fraction of particles exceeding this threshold. Thus, our experimental validation directly addresses the practical relevance of Li‐rich NCM materials.

This alignment between predictive accuracy and probabilistic confidence is critical for risk‐aware decision‐making, enabling direct use of model outputs to guide synthesis optimization. By combining chemistry‐aware imputation to maximize usable data, robust cross‐validation to prevent overfitting, and UQ to communicate predictive reliability, the framework demonstrates that accurate and trustworthy predictions are achievable even in data‐sparse, high‐variability materials design problems. Stable train–test performance, successful OOD validation in three of four cases, effective use of the 293‐sample imputed‐missing target dataset, and well‐calibrated probabilistic outputs confirm the pipeline's ability to deliver high accuracy under challenging synthesis conditions.

Its scalability to handle incomplete datasets and the provision of uncertainty estimates make it well‐suited for extension to multi‐output prediction, such as jointly tailoring primary particle size and electrochemical performance or modeling complex co‐doping effects. While the present framework focuses on mean primary particle size prediction, extending it to particle‐size distributions through distributional or quantile regression could capture both central tendency and variability, providing a more comprehensive picture of microstructural morphology. Such an extension, however, will require larger datasets with standardized per‐particle statistics, which remain scarce in the current literature.

Model interpretation via SHAP analysis revealed a feature hierarchy consistent with physical understanding. The second sintering temperature emerged as the most influential predictor, with higher values consistently driving larger primary particle sizes, in line with prior studies attributing this to enhanced atomic mobility and grain coarsening at elevated temperatures.^[^ ^25^, ^26^, ^27^, ^28^ ^]^ The first sintering time also contributed positively, though less strongly; for example, extending the first sintering step can promote coarsening without severely affecting structural integrity or morphology.^[^ ^28^ ^]^ By contrast, compositional variables had a negligible influence. For instance, NMC811 and LiNi_0.9_Mn_0.05_Co_0.05_O_2_ (NMC90), synthesized under identical co‐precipitation and calcination conditions, exhibited nearly identical mean particle sizes (≈ 500  ± 50 nm) despite a 10% Ni content difference.^[^ ^29^ ^]^ Likewise, molten‐salt‐synthesized NMC811 and NMC622 differed only slightly in size (6.4 µm vs 7.0 µm) under equivalent flux conditions.^[^ ^30^ ^]^ Together, these findings confirm that processing conditions—particularly sintering parameters—are more decisive than stoichiometry in governing the microstructural evolution of NCM materials.

The compositional fractions and sintering parameters analyzed in this study were chosen because they are consistently reported in the literature, enabling a uniform dataset while avoiding systematic gaps caused by sparsely documented variables. Nevertheless, other synthesis factors, such as precursor particle size and reactants, can also influence particle growth and morphology,^[^ ^31^ ^]^ but are rarely available in published reports and were therefore treated here as unobserved. Incorporating such variables through systematic data collection or controlled experiments would represent an important direction for future work.

Finally, to position our work in a broader perspective, **Table** **4** compares representative small‐dataset ML studies in materials science with respect to dataset size, problem type, model choice, the use of probabilistic UQ and calibration, and the presence of external validation. Because prediction targets and metrics differ across studies, direct numerical comparisons are not meaningful; instead, the table emphasizes methodological capabilities. As shown in Table 4, these comparative examples—from bentonite thermal conductivity prediction with Gaussian Process Regression (GPR), to early‐cycle battery life forecasting, small‐data crude estimation of property augmentation, and explicit UQ‐driven modeling—demonstrate that coupling domain‐specific knowledge or augmentation strategies with UQ can significantly improve model robustness and trustworthiness in data‐sparse regimes. Small datasets are common in this field, often leading to overfitting, poor generalization, and limited external validation.^[^ ^35^ ^]^ Prior studies have addressed these challenges through feature engineering, data augmentation, or predictive modeling.^[^ ^36^ ^]^ However, few have combined these elements within a single framework. In contrast, our approach expands the usable training set through chemistry‐aware MatImpute (+ 105% increase from 143 to 293 entries), achieves calibrated probabilistic predictions on a complete held‐out test set, and uniquely validates performance on OOD experimental samples, where three of four NCM811 cases exhibited normalized errors near unity. Together, these contributions demonstrate that trustworthy predictions are achievable even in data‐sparse, high‐variability settings when domain‐specific knowledge is combined with explicit uncertainty quantification.

**Table 4:** Representative small‐dataset ML studies in materials science, compared by dataset size, problem type, UQ, calibration, and OOD validation.

Study	Dataset size	Problem type	ML models	Probabilistic UQ and calibration	Metric performance	External validation
Zhang & Ling (2018)^[^ ^32^ ^]^	108	Bandgap, lattice thermal conductivity, and elastic properties	Kernel ridge regression, gradient boosting	Not UQ‐focused, but addressed small‐data robustness using crude estimation of property to reduce bias	Scaled error reductions	No
Bang et al. (2020)^[^ ^33^ ^]^	176	Predicting thermal conductivity of compacted bentonite	Various ML models (linear regression, decision tree, support vector machine, Gaussian process, ensemble)	No	Best RMSE = 0.072–0.073 (XGBoost & GPR)	No
Severson et al. (2019)^[^ ^34^ ^]^	124	Early prediction of LIB cycle life	Feature‐based elastic net regression	No explicit probabilistic output, but tested with an independent secondary dataset	9.1% test error (regression, 4.9% test error (classification)	No
Avula et al. (2022)^[^ ^35^ ^]^	273	Predicting shear viscosity of Lennard–Jones fluid	Gaussian Process Regression, ensemble models	Explicit UQ via GPR predictive variance and ensemble spread; applicability domain mapping	Demonstrated UQ‐guided filtering improved reliability; reduced out‐of‐domain prediction risk	No
This study	293 (with imputed missing targets)/ 143 (without missing target values)	Predicting mean primary particle size of Li‐rich NCM materials	NGBoost with chemistry‐aware imputation	Probabilistic modeling (NGBoost output distributions)	R^2^ = 0.866, NLL = –1.59 on held‐out test set, uncertainty calibration AUCE = 0.133	Yes (experimental NCM811; normalized error near unity for three out of four samples)

## 4 Conclusion

Coupling chemistry‐aware imputation with a probabilistic NGBoost regressor enables calibrated, data‐efficient prediction of mean primary particle size in Li‐rich NCM materials. Expanding training from 143 fully reported rows to 293 entries via missing target value imputation yielded strong held‐out performance (R^2^ ≈ 0.866; AUCE ≈ 0.133), with MatImpute providing the best accuracy–calibration trade‐off. SHAP analyses consistently indicated that sintering processing, especially the second sintering temperature and the first sintering time, dominates over composition within the studied range. Out‐of‐distribution validation on four NCM811 samples showed small absolute errors (≤ 0.13 µm in three cases) and normalized errors near unity when combining experimental and predictive dispersion, indicating appropriately scaled uncertainty. Limitations arise from heterogeneous literature reporting (scarce per‐entry variances, non‐standardized imaging) and a modest experimental set; these are mitigated by distributional scoring (NLL, AUCE) and an error normalization that integrates measured and predictive dispersion. Future work will develop distribution‐aware models (e.g., distributional or quantile regression) to predict particle size distributions once standardized per‐particle statistics become available.

## 5 Experimental Section

### The NGBoost Training and Test

The main dataset of Li‐rich NCM materials was curated manually from the literature by the previous research,^[^ ^15^ ^]^ while in the present study, it was augmented with additional entries obtained through human‐assisted Generative Pre‐trained Transformer (GPT) extraction, all of which were subsequently verified by human review.

Each entry contained synthesis parameters (first/second sintering temperatures and times), compositional fractions (Li in the TM layer, Ni, Mn, and Co), and the primary particle size. When particle sizes were reported as ranges, only those cases were converted to mean values by averaging the bounds and expressing them in micrometers (µm). Missing values were present in both input and target variables due to inconsistent reporting. To address this, four imputation methods—MatImpute, KNN, MIC, and Mean—were applied. Two workflows were adopted for missing data handling: i) imputing missing target values (y) along with inputs (X), thereby retaining incomplete entries (resulting in a dataset called imputed X–y dataset), and ii) discarding entries with missing targets (y) prior to imputation, yielding a smaller dataset (resulting in a dataset called imputed X‐dataset).

Consistent with the two missing‐data workflows, NGBoost was trained^[^ ^24^ ^]^ under two strategies. In the first strategy, entries with missing targets were retained after imputation (293 training entries; ≈81%), and evaluation was performed on a fully observed held‐out test set (70 entries; ≈19%). In the second strategy, rows with missing targets were removed prior to imputation (143 training entries; ≈67%) while using the same 70‐entry fully observed test set (≈33%). In both strategies, the test set contained no missing values (inputs or targets) and was excluded from imputation and model fitting to ensure an unbiased generalization assessment.

Before modeling, input features (Li fraction in the TM layer; Ni, Co, and Mn fractions; first and second sintering temperatures and times) were transformed using a PowerTransformer with the Yeo–Johnson family (https://scikit‐learn.org/stable/modules/generated/sklearn.preprocessing.PowerTransformer.html). To prevent information leakage, the transformer was fit on each training fold only and then applied to the corresponding validation or test split.

NGBoost was implemented as a probabilistic regressor configured with a Gaussian (Normal) output distribution and a decision‐tree base learner.^[^ ^24^ ^]^ For reproducibility, the learner and ensemble were initialized with a fixed random seed. Training proceeded for five thousand boosting iterations with a learning rate of 0.00999999999. Early stopping was governed by an internal validation split comprising fifteen percent of the training data. At each boosting step, both row‐wise and column‐wise subsampling were employed, each at fifty percent, and recorded training progress verbosely. Model selection followed a 10‐fold cross‐validation protocol with shuffled partitions and a fixed seed to ensure comparability across folds.

Predictive performance on the held‐out test set was quantified by the R^2^ for accuracy and NLL for probabilistic fit, while calibration was assessed by the AUCE computed from empirical coverage versus predicted quantiles (101 levels). For interpretability, SHAP values were computed to obtain global feature rankings and local attributions linking synthesis parameters to the primary particle‐size predictions (https://github.com/dsgibbons/shap/blob/master/docs/index.rst).

All experiments were run on an NVIDIA GeForce RTX 4080 using Python 3.12.11 with ngboost 0.5.6; the chemistry‐aware MatImpute implementation was obtained from the MatImpute repository (https://github.com/big‐material/MatImpute).^[^ ^16^ ^]^

### Experimental Validation

To evaluate the predictive reliability and generalizability of the NGBoost prediction trained on the MatImpute‐imputed *X*−*y* dataset with imputed missing target values, four NCM811 samples were synthesized under a range of sintering parameters and compositional conditions. These samples underwent experimental synthesis and particle characterization. Although the model was initially trained on Li‐rich NCM materials, it was validated using NCM811 compositions with a zero lithium fraction in the transition metal layer. This validation set was deliberately selected to evaluate the model's ability to extrapolate to data distributions that differ significantly from those seen during training. In particular, it was designed to assess the model's performance under OOD conditions.

The NCM811 samples were generated using a custom in‐house Bayesian Optimization framework designed to maximize the initial discharge capacity. This framework randomly proposes synthesis conditions—including precursor selection, hydrothermal treatment, and sintering parameters—based on the defined optimization objective. While the optimization routine itself was beyond the scope of this study, interested readers may contact the authors for access to the source code.

The four NCM811 samples generated through the Bayesian Optimization process were designated as NCM811_1, NCM811_2, NCM811_3, and NCM811_4, each synthesized under a distinct combination of precursor chemistries. Specifically, NCM811_1 was synthesized using LiOH·H_2_O, NiSO_4_·6H_2_O, CoSO_4_·7H_2_O, and Mn(CH_3_COO)_2_·4H_2_O. NCM811_2 employed LiOH·H_2_O, Ni(CH_3_COO)_2_·4H_2_O, CoSO_4_·7H_2_O, and Mn(CH_3_COO)_2_·4H_2_O. NCM811_3 utilized LiOH·H_2_O, NiSO_4_·6H_2_O, Co(CH_3_COO)_2_·4H_2_O, and Mn(CH_3_COO)_2_·4H_2_O, while NCM811_4 was prepared with LiOH·H_2_O, Ni(CH_3_COO)_2_, Co(CH_3_COO)_2_·4H_2_O, and Mn(CH_3_COO)_2_·4H_2_O. The full sintering conditions for each sample, including temperature (T) and duration (t), are summarized in **Table** **5**.

**Table 5:** Sintering conditions for each sample of NCM811.

Sample	Hydrothermal T [°C]	Hydrothermal t [h]	1st Sinter. T [°C]	1st Sinter. T [h]	2nd Sinter. T [°C]	2nd Sinter. t [h]
NCM811_1	183	9	469	4	750	26
NCM811_2	200	11	451	4	761	20
NCM811_3	199	12	483	3	744	6
NCM811_4	200	11	476	5	741	9

A hydrothermal method was used to synthesize NCM811 precursors. Stoichiometric amounts of manganese, cobalt, and nickel acetates or sulfates (Sigma–Aldrich) were dissolved in deionized water, with CO(NH_2_)_2_ (Junsei) added as a precipitating agent. This solution was subjected to hydrothermal treatment for each temperature and time condition. The precipitated precursors were thoroughly washed through vacuum filtration with ethanol several times. The washed materials were mixed with lithium sources, pelletized, and sintered in the given temperature and time parameters. A 3% excess of lithium source was added to the samples to compensate for lithium evaporation.

Morphological and structural characterization was conducted using scanning electron microscopy (SEM; SU5000, Hitachi) and X‐ray diffraction (XRD; SmartLab, Rigaku). XRD measurements were taken across a 2θ range of 10–80° with a step size of 0.01° to confirm phase purity and crystal structure. Primary particle sizes were extracted from SEM images using ImageJ software (https://imagej.net/ij/). For each particle, two orthogonal diameters were measured by drawing horizontal and vertical lines from edge to edge across the particle boundary. The average of these two diameters was taken as the particle size, which reduces bias arising from anisotropic or irregular particle shapes. This procedure was repeated across a statistically representative number of particles in each sample, and the mean primary particle size was computed by averaging these measurements.

The resulting mean values were used as external benchmarks to evaluate model predictions under OOD conditions. For the four synthesized NCM811 samples, both the mean and standard deviation of the measured particle sizes were reported. To enable a statistically consistent comparison between experimental observations and probabilistic model outputs, a normalized error (Equation S1, Supporting Information) was calculated by combining experimental variance with model‐predicted uncertainty.

## Conflict of Interest

The authors declare no conflict of interest.

## Supporting information



Supporting Information

## Data Availability

The full dataset will be made available upon reasonable request. In addition, we provide a DOI for each data point in our GitHub repository (https://github.com/MIIMSEKAIST/NGBoost_Li_rich_NCM_oxide). This repository also contains the SEM images, the particle sizes extracted from these images, and the synthesis parameters used for the experimental validation recommended by our in‐house Bayesian Optimization framework.
