# Supplementary material for: Uncertainty‐Quantified Primary Particle Size Prediction in Li‐Rich NCM Materials via Machine Learning and Chemistry‐Aware Imputation
Source: Adv Sci (Weinh). 2025 Oct 8;13(2):e15694. doi: 10.1002/advs.202515694 (PMC12786289; doi:10.1002/advs.202515694)
Supplement: Supplementary file 1 — Supporting Information [file ADVS-13-e15694-s001.docx]

Supporting Information

Uncertainty-Quantified Primary Particle Size Prediction in Li-Rich NCM Materials via Machine Learning and Chemistry-Aware Imputation

Benediktus Madika^1^, Chaeyul Kang^1^, JooSung Shim^1^, Taemin Park^1^, Jung Hyeon Moon^1^, EunAe Cho^1^, Seungbum Hong^1,2*^

^1^Department of Materials Science and Engineering, KAIST, Daejeon 34141, Korea

^2^KAIST Institute for NanoCentury (KINC), KAIST, Daejeon, 34141, Korea

E-mail:[seungbum@kaist.ac.kr](mailto:seungbum@kaist.ac.kr)

**Table S1**. The NLL values for each fold of the NGBoost model trained on the imputed $X$ datasets.

| Imputed $X$ Datasets | Fold | | | | | | | | | |  |
| --- | --- | --- | --- | --- | --- | --- | --- | --- | --- | --- | --- |
|  | 1 | 2 | 3 | 4 | 5 | 6 | 7 | 8 | 9 | 10 | Avg NLL |
| MatImpute-imputed dataset | 1.22 | -0.16 | 3.66 | 3.80 | 0.32 | 1.09 | -0.56 | 0.44 | 0.31 | -0.18 | 0.99 |
| KNN-imputed dataset | 1.22 | 0.32 | 3.66 | 3.27 | 0.33 | 1.09 | -0.69 | 0.49 | 0.33 | -0.32 | 0.97 |
| MICE-imputed dataset | 1.22 | 0.311 | 3.66 | 2.99 | 0.29 | 1.09 | -0.33 | 0.43 | 0.36 | -0.05 | 1.00 |
| Mean-imputed dataset | 1.22 | 0.02 | 3.66 | 2.71 | 0.23 | 1.09 | -0.55 | 0.47 | 0.33 | -0.28 | 0.89 |

**Table S2**. The R^2^ values for each fold of the NGBoost model trained on imputed $X$ datasets.

| Imputed $X$ Datasets | Fold | | | | | | | | | | |
| --- | --- | --- | --- | --- | --- | --- | --- | --- | --- | --- | --- |
|  | 1 | 2 | 3 | 4 | 5 | 6 | 7 | 8 | 9 | 10 | Avg R^2^ |
| MatImpute-imputed dataset | -13.5 | 0.96 | -0.17 | -0.35 | -0.89 | -1.45 | 0.98 | 0.60 | 0.94 | 0.54 | -1.23 |
| KNN-imputed dataset | -13.5 | 0.90 | -0.17 | -0.37 | -0.94 | -1.33 | 0.99 | 0.59 | 0.94 | 0.57 | -1.23 |
| MICE-imputed dataset | -14.0 | 0.93 | -0.17 | -0.39 | -0.74 | -1.32 | 0.98 | 0.59 | 0.93 | 0.50 | -1.27 |
| Mean-imputed dataset | -14.0 | 0.94 | -0.17 | -0.35 | -0.96 | -1.32 | 0.99 | 0.62 | 0.94 | 0.59 | -1.27 |

**Table S3**. The R^2^ cross-validation of the NGBoost trained on the 143 data entries randomly drawn from the 293-entry imputed $X-y$ datasets.

| Imputed $X-y$ Datasets | Avg R^2^ | Avg NLL |
| --- | --- | --- |
| MatImpute-imputed dataset | -8.20 | 0.67 |
| KNN-imputed dataset | -5.27 | 1.57 |
| MICE-imputed dataset | -0.04 | 0.71 |
| Mean-imputed dataset | -3.20 | 0.55 |


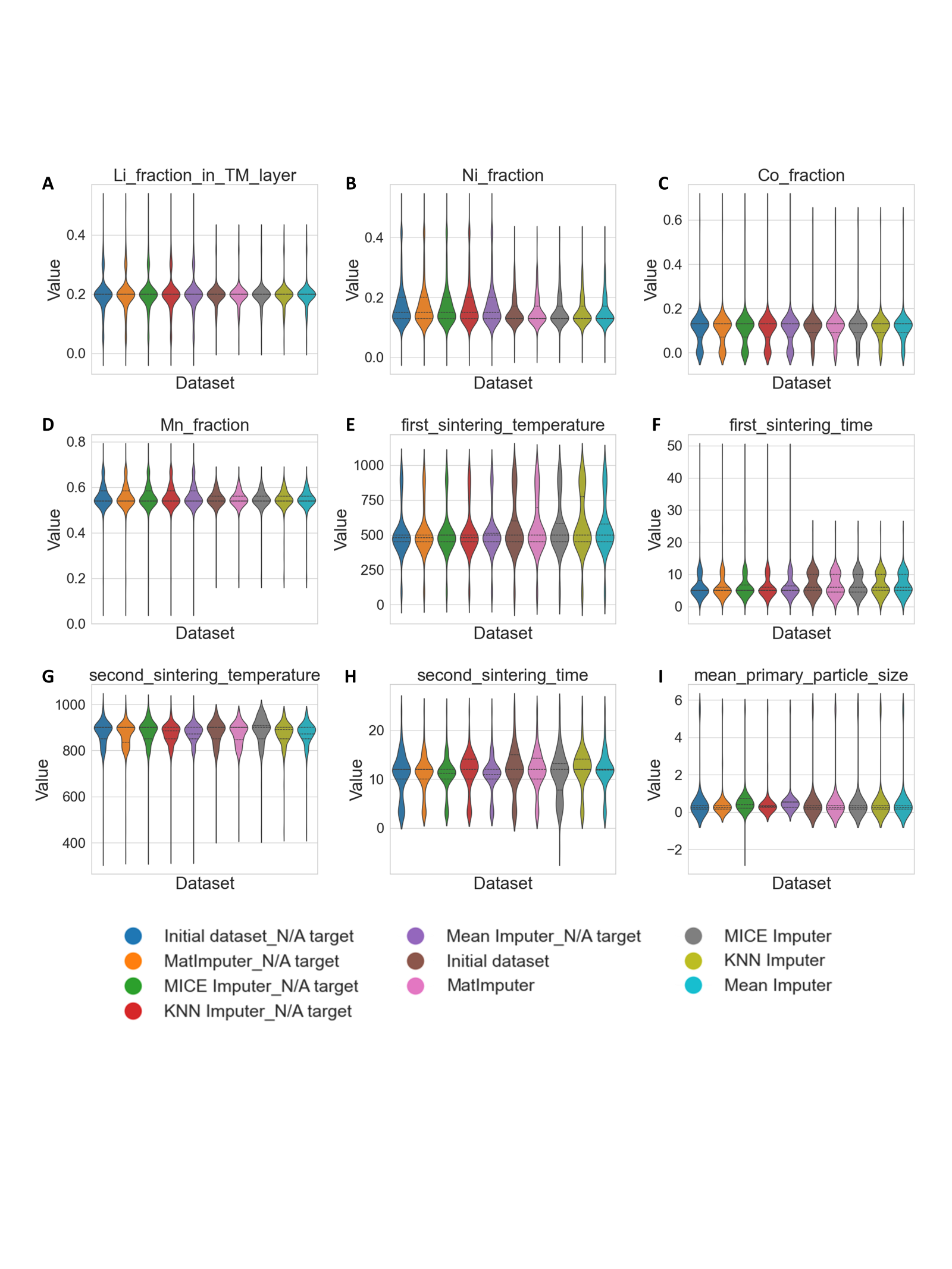


**Figure S1.** Violin plots comparing the distributions of features before and after imputation using different methods. The solid line represents the median, while the dashed line shows the interquartile range (IQR). The upper and lower tails indicate the range of values outside the IQR. Long tails, whether positive or negative, suggest the presence of outliers or high variability in the data. A long negative tail may indicate the imputation of extreme low values, while a long positive tail suggests high positive values. The width of the violin indicates data density, with wider sections showing higher concentrations of data. Stoichiometric features, having no missing data, show consistent distributions. In contrast, processing parameters and mean primary particle size, with missing data, exhibit more variability in the imputed datasets. MatImpute best preserves the original data distribution, showing slight shifts in tails and minimal disruption to the central trends. In contrast, other methods like KNN and MICE lead to wider distributions with unrealistic imputations, especially in the tails, reflecting significant distortion in the data.


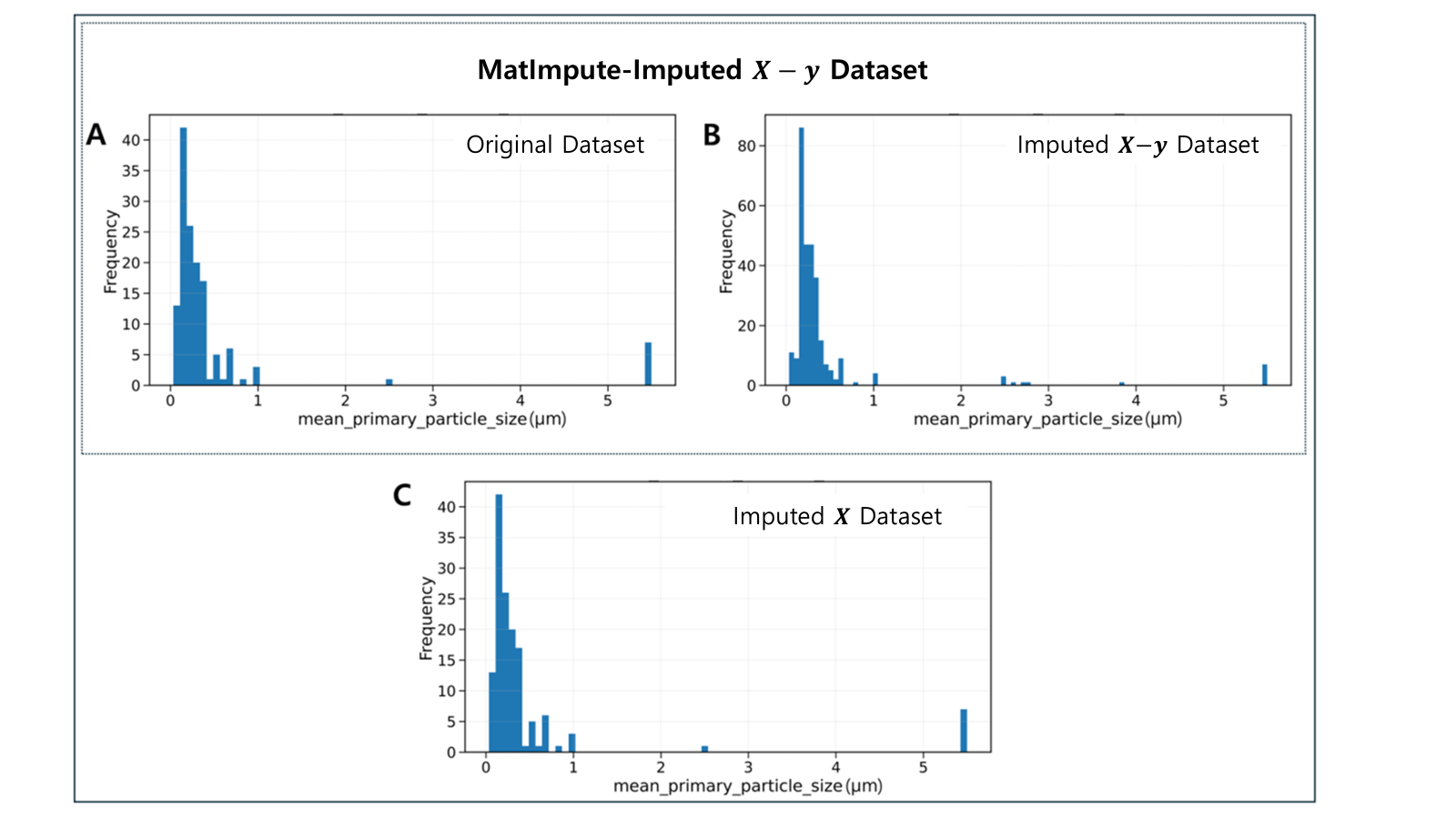


**Figure S2**. Distributions of reported and imputed mean primary particle sizes. (A) Original reported targets show a right-skewed distribution dominated by sub-micron values with a sparse upper tail. (B) After MatImpute imputation, the overall shape is preserved, with the imputed missing target value (imputed $X-y$ dataset) filling the sub-micron bulk and no artificial extremes introduced. (C) The imputed $X$dataset retains the central trend but exhibits reduced coverage due to list-wise deletion.


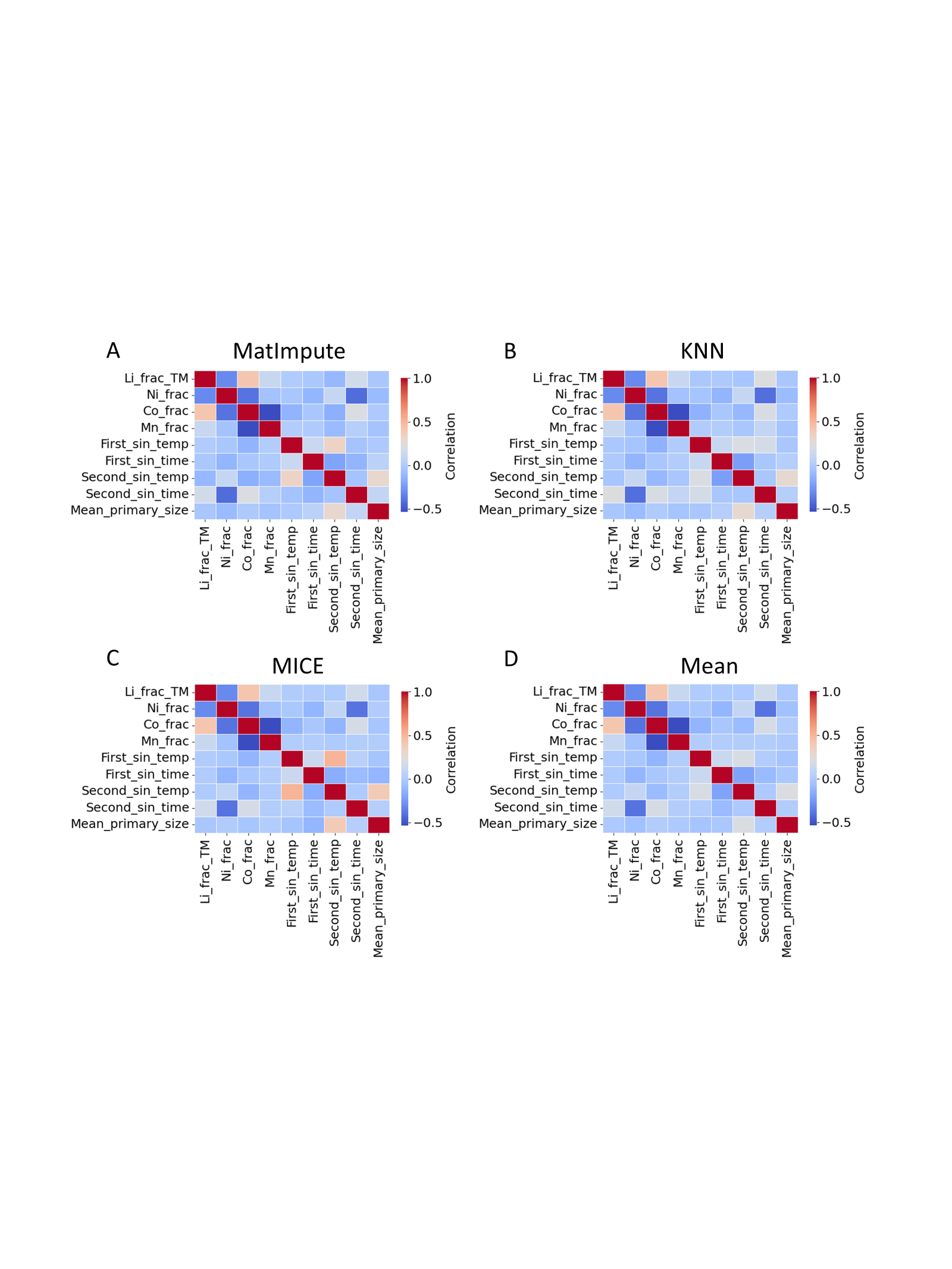


**Figure S3**. Feature correlations of the training data with imputed missing target value. The heatmaps represent the Pearson correlation coefficients between the features, including various fractions and sintering parameters, and the mean primary particle size. Positive correlations are shown in red, while negative correlations are shown in blue, with the color intensity indicating the strength of the correlation. All imputation methods (MatImpute, KNN, MICE, and Mean) result in nearly identical correlation patterns across the features, suggesting that these methods preserve the underlying relationships in the data. This indicates that, for this dataset, the choice of imputation method does not significantly affect the feature correlations, reflecting robustness in the imputation techniques used.


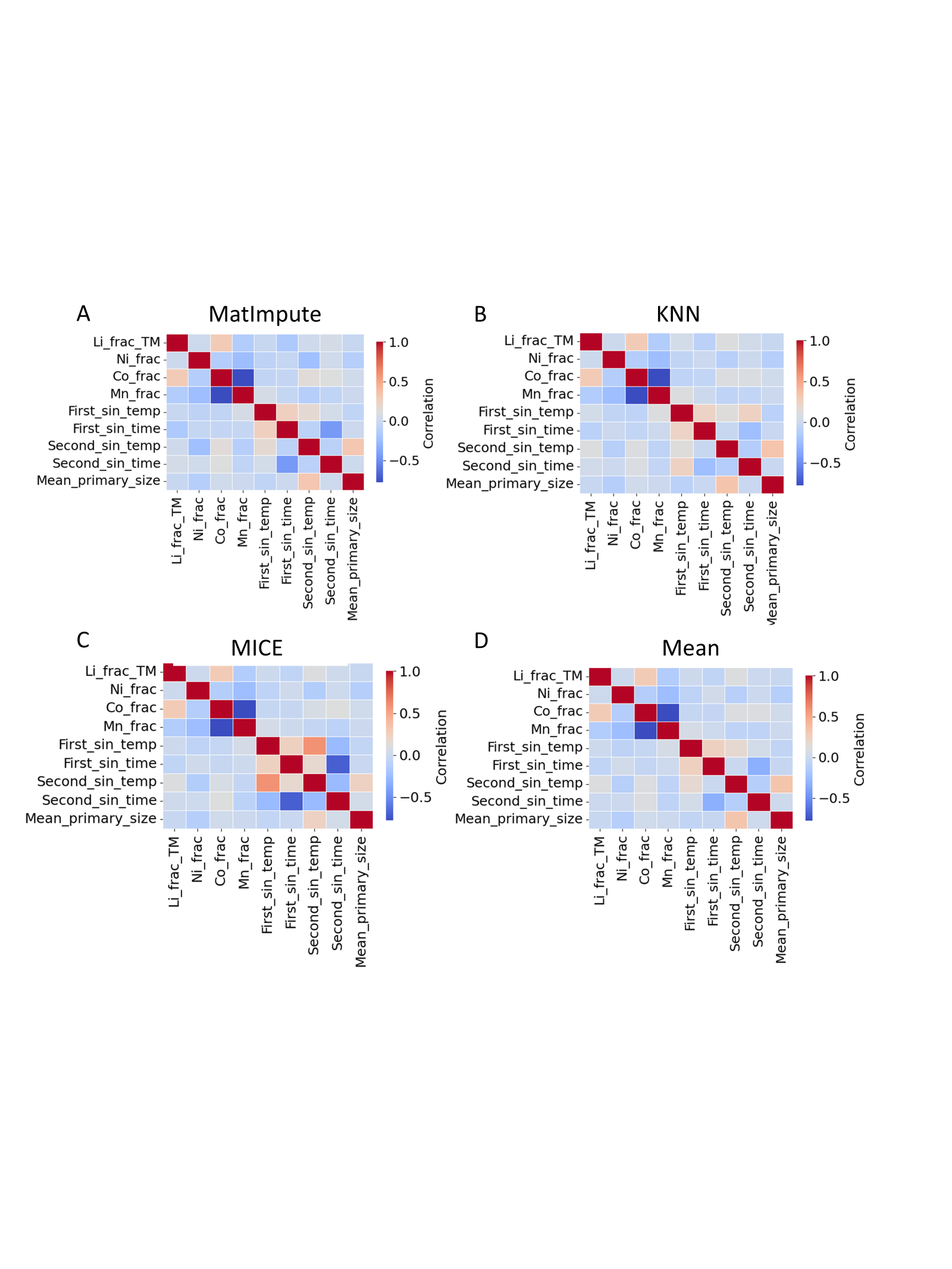


**Figure S4**. Feature correlations of the training data without imputed missing target value. The heatmaps show the Pearson correlation coefficients between various features, including elemental fractions, sintering parameters, and mean primary particle size. The correlation structures remain consistent across all imputation methods (MatImpute, KNN, MICE, and Mean), demonstrating that the imputation methods do not significantly alter the feature relationships. Positive correlations are represented in red, while negative correlations are shown in blue, with the color intensity indicating the strength of the correlation.

**
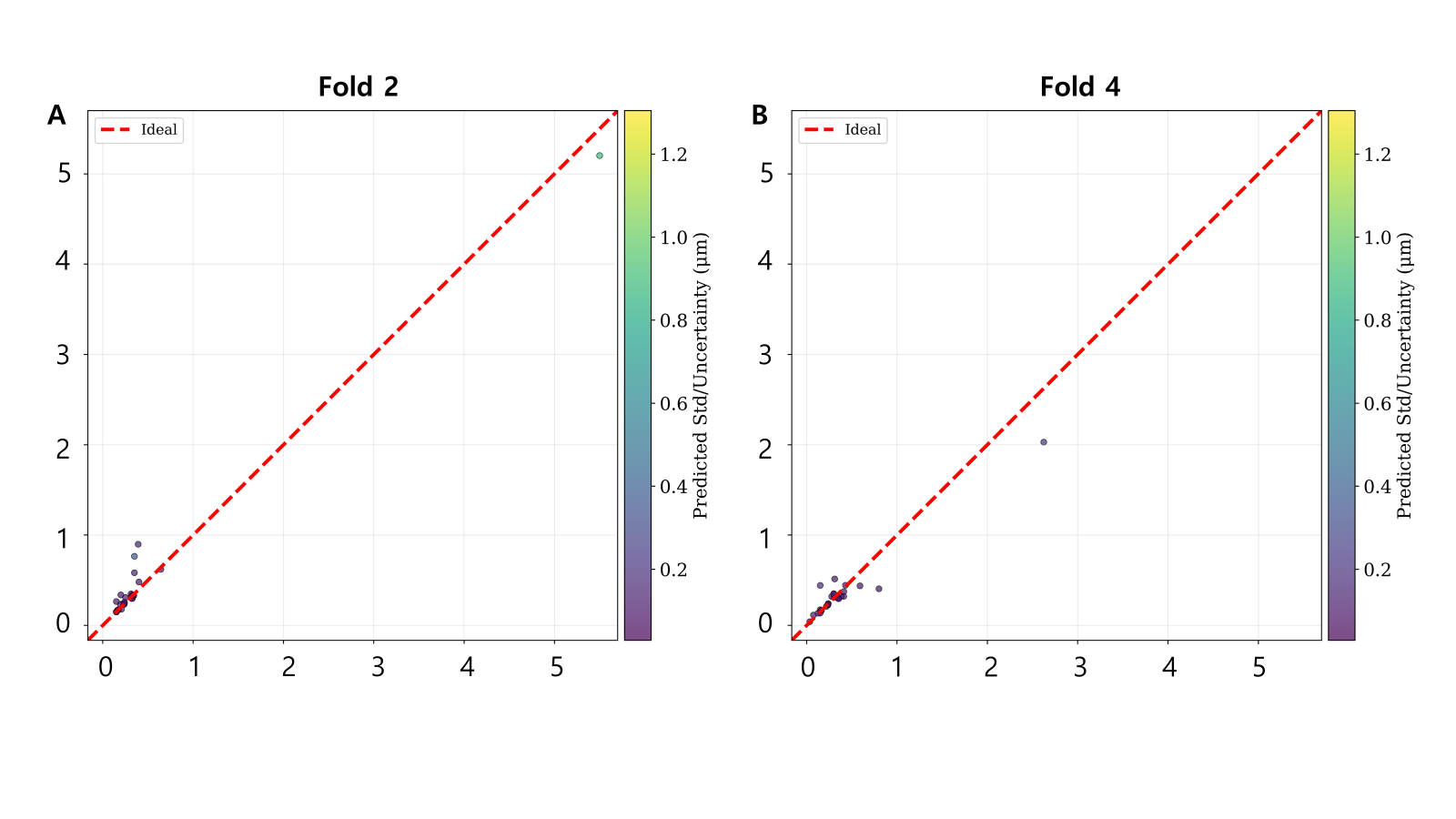
**

**Figure S5**. Representative parity plots selected from the remaining cross-validation runs. A) Fold 2 (R² = 0.98, NLL = -0.82) and B) Fold 4 (R² = 0.88, NLL = -0.64).


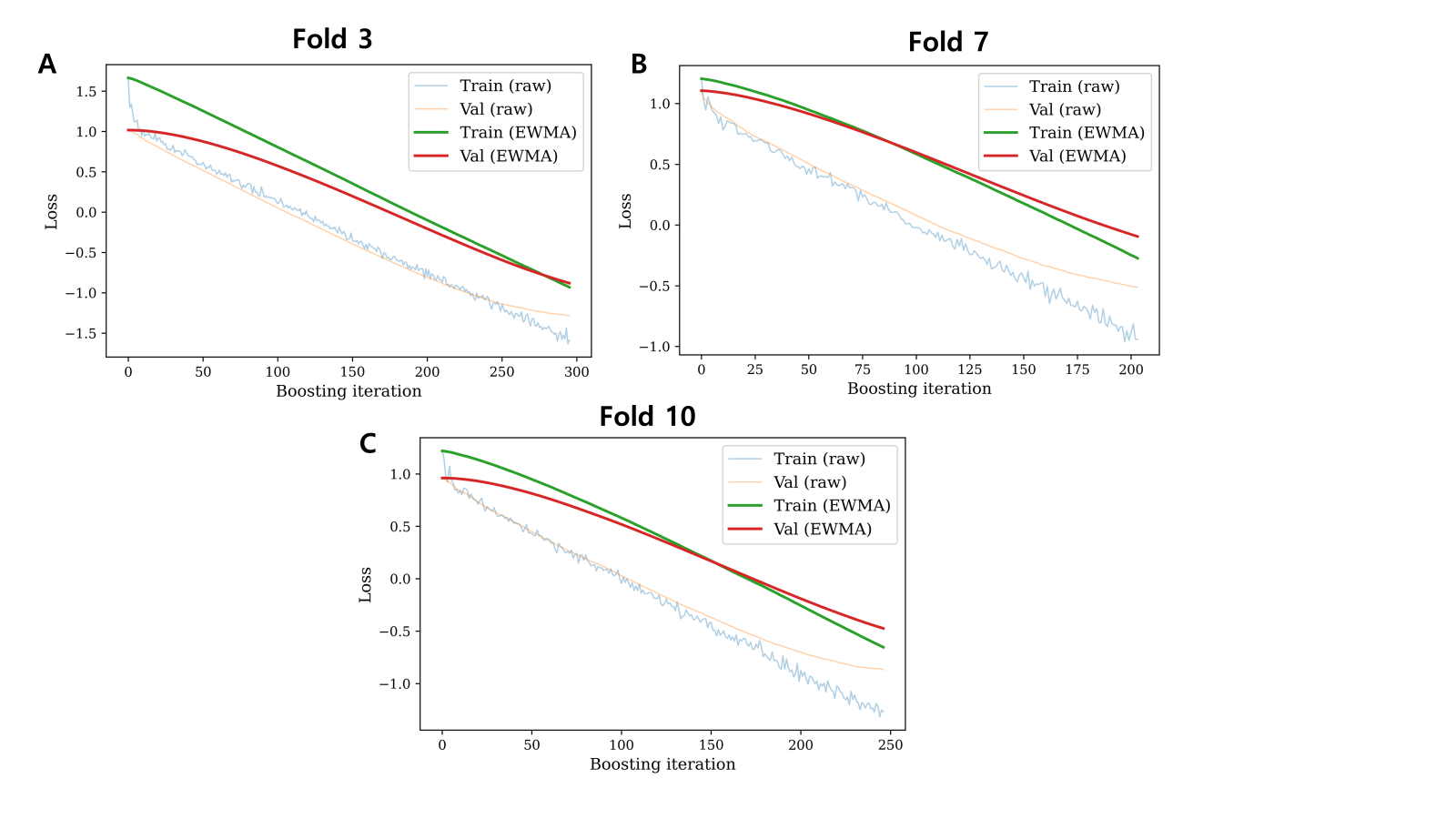


**Figure S6**. Training-loss trajectories for NGBoost across three cross-validation folds. The x-axis denotes boosting iteration (one additional weak learner added per iteration). Thin curves are raw logged losses; thick curves are EWMA-smoothed trends (span = 150) to emphasize the underlying dynamics. A) Fold 3: Both training and validation losses decrease monotonically with a small, stable generalization gap and the lowest terminal validation loss—best performance among the three, with no evidence of late-stage divergence. B) Fold 7: Loss decreases more gradually, and early stopping occurs sooner; the train–validation gap is larger, and the terminal validation loss is the weakest of the three, though no rebound indicative of overfitting is observed. C) Fold 10: Smooth concurrent decline of training and validation losses with a modest, stable gap; terminal validation loss is intermediate—second best overall.


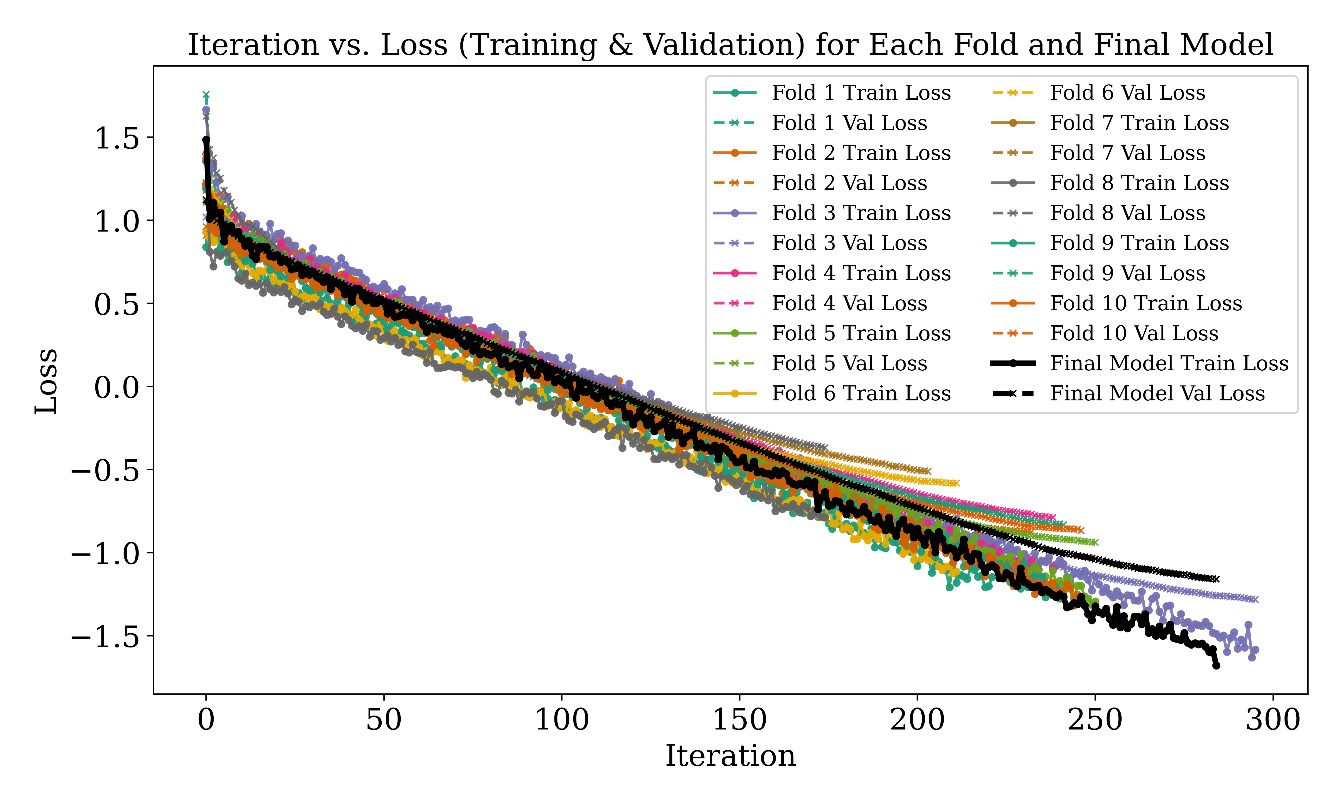


**Figure S7**. Training and validation loss trajectories (NLL) for each fold (1–10) and for the final NGBoost model trained on the MatImpute-imputed $X-y$ dataset. Loss is logged at every boosting iteration up to 300, with solid lines representing training loss and dashed lines representing validation loss. Several folds terminate before the maximum iteration due to early stopping once the validation loss plateaus. In all cases, validation losses decrease, while train–validation gaps remain modest, indicating effective learning with minimal overfitting. The final model shows convergence behavior consistent with that observed across the cross-validation folds.


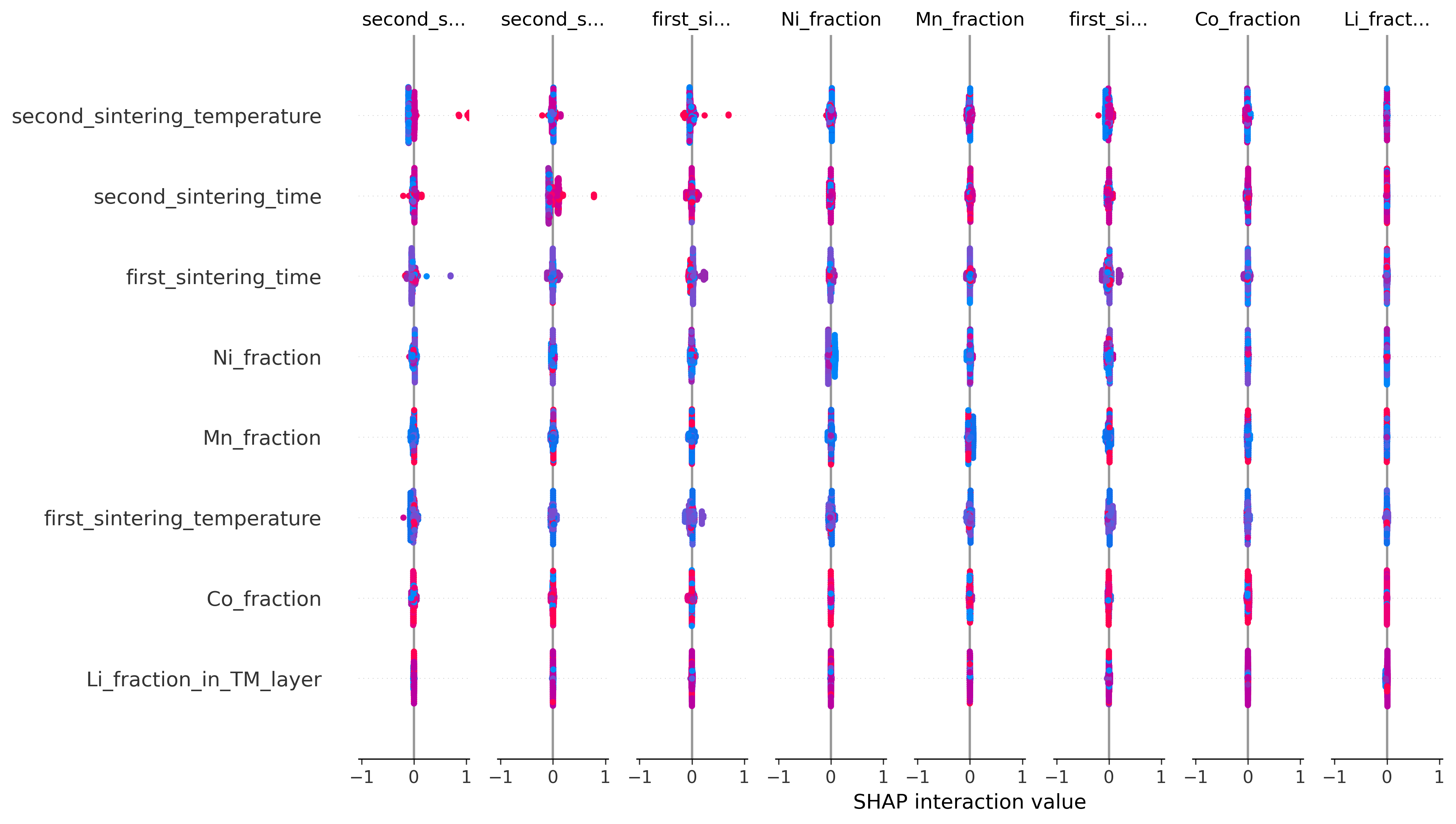


**Figure S8**. SHAP interaction summary for the NGBoost model (MatInpute‑imputed $X-y$). Columns denote *source* features and rows denote *target* features. Each point is a sample; the x-axis is the SHAP interaction value (pairwise contribution beyond the sum of main effects). Color encodes the source feature value (red = high, blue = low). A broader horizontal spread indicates a stronger interaction. The strongest interactions are observed among the sintering variables, particularly between the second sintering temperature and the first/second sintering times, consistent with coarsening being controlled by the combined temperature–time thermal budget; interactions involving Ni/Co/Mn/Li fractions are comparatively weak.

**Equation S1:**

Normalized error ($\boldsymbol{\epsilon}_{\boldsymbol{z}}$)computation:

$$\boldsymbol{\sigma}_{\boldsymbol{com}\boldsymbol{bined}}\boldsymbol{=}\sqrt{\boldsymbol{\sigma}_{\boldsymbol{m}}^{\boldsymbol{2}}\boldsymbol{+}\boldsymbol{\sigma}_{\boldsymbol{p}}^{\boldsymbol{2}}}$$

$$\boldsymbol{\epsilon}_{\boldsymbol{z}}\boldsymbol{=}\frac{\boldsymbol{\mu}_{\boldsymbol{p}}\boldsymbol{-}\boldsymbol{\mu}_{\boldsymbol{m}}}{\boldsymbol{\sigma}_{\boldsymbol{com}}}$$

Where m is measured, p is predicted.
